# The Use of Immune Checkpoint Inhibitors in Oncology and the Occurrence of AKI: Where Do We Stand?

**DOI:** 10.3389/fimmu.2020.574271

**Published:** 2020-10-08

**Authors:** Rossana Franzin, Giuseppe Stefano Netti, Federica Spadaccino, Camillo Porta, Loreto Gesualdo, Giovanni Stallone, Giuseppe Castellano, Elena Ranieri

**Affiliations:** ^1^ Nephrology, Dialysis and Transplantation Unit, Department of Emergency and Organ Transplantation, University of Bari, Bari, Italy; ^2^ Clinical Pathology, Center of Molecular Medicine, Department of Medical and Surgical Sciences, University of Foggia, Foggia, Italy; ^3^ Oncology, Department of Biomedical Sciences and Human Oncology, University of Bari Aldo Moro, Bari, Italy; ^4^ Nephrology, Dialysis and Transplantation Unit, Department of Medical and Surgical Sciences, University of Foggia, Foggia, Italy

**Keywords:** immune checkpoint inhibitors, AKI (acute kidney injury), mTOR inhibitor, CTLA-4, PD-1-PDL-1 axis, immunosenescence and inflammaging, gut microbiome, renal cell cancer (RCC)

## Abstract

Immune checkpoint inhibitors (ICIs) are a novel class of immunotherapy drugs that have improved the treatment of a broad spectrum of cancers as metastatic melanoma, non-small lung cancer or renal cell carcinoma. These humanized monoclonal antibodies target inhibitory receptors (e.g. CTLA-4, PD-1, LAG-3, TIM-3) and ligands (PD-L1) expressed on T lymphocytes, antigen presenting cells and tumor cells and elicit an anti-tumor response by stimulating immune system. Nevertheless, the improved overall survival is complicated by the manifestation of Immune-related Adverse Effects (irAEs). During treatment with ICIs, the most common adverse kidney effect is represented by the development of acute kidney injury (AKI) with the acute tubulointerstitial nephritis as recurrent histological feature. The mechanisms involved in ICIs-induced AKI include the re-activation of effector T cells previously stimulated by nephrotoxic drugs (i.e. by antibiotics), the loss of tolerance versus self-renal antigens, the increased PD-L1 expression by tubular cells or the establishment of a pro-inflammatory milieu with the release of self-reactive antibodies. For renal transplant recipient treated with ICIs, the increased incidence of rejection is a serious concern. Therefore, the combination of ICIs with mTOR inhibitors represents an emerging strategy. Finally, it is relevant to anticipate which patients under ICIs would experience severe irAEs and from a kidney perspective, to predict patients with higher risk of AKI. Here, we provide a detailed overview of ICIs-related nephrotoxicity and the recently described multicenter studies. Several factors have been reported as biomarkers of ICIs-irAEs, in this review we speculate on potential biomarkers for ICIs-associated AKI.

## Introduction

Cancer immunotherapy encompasses a number of different treatments aimed at stimulating the immune system in order to promote the recognition and the elimination of tumor cells (1). In the past decade, Immune checkpoints inhibitors (ICIs) have emerged as anticancer agents able to modify, for the better, the natural history of a wide range of malignancies, such as melanoma, renal cell carcinoma, non-small cell lung cancer (NSCLC), bladder cancers, Hodgkin lymphoma and others (2). Although these agents have dramatically improved the prognosis of many cancer patients, they are critically associated with a broad spectrum of sometimes ill-defined adverse events, caused by the uncontrolled activation of the immune system, due to the lack of physiological brakes (i.e. the immune checkpoints themselves), referred to as immune-related adverse events (irAEs) characterized by clinical manifestations that closely resemble autoimmunity disorders (3, 4). Given the extrarenal clearance of ICIs, the contribution of these agents to kidney toxicity has been neglected and underestimated for several years (5). This was further complicated by the fact too often, renal toxicities from anticancer agents in general, are reported, within oncology clinical trials, just as “creatinine increase”, or similar definitions, without any further pathogenic insight. On the contrary, increasing evidence supports the central involvement of ICIs in the development of acute kidney injury (AKI), proteinuria, and renal electrolyte abnormalities. Strikingly, after episodes of ICIs-induced AKI, impaired renal function recovery correlated with increased mortality (6). In this review, we will discuss the molecular pathways modulated by ICIs on T-cell activation, the proposed mechanisms of ICIs-related renal injury, with a particular focus on the development of AKI, as well as recent insights into clinical trials, and biomarkers studies aimed at assessing response to treatment.

## Immune Checkpoint on T Lymphocytes

**Figure 1:**
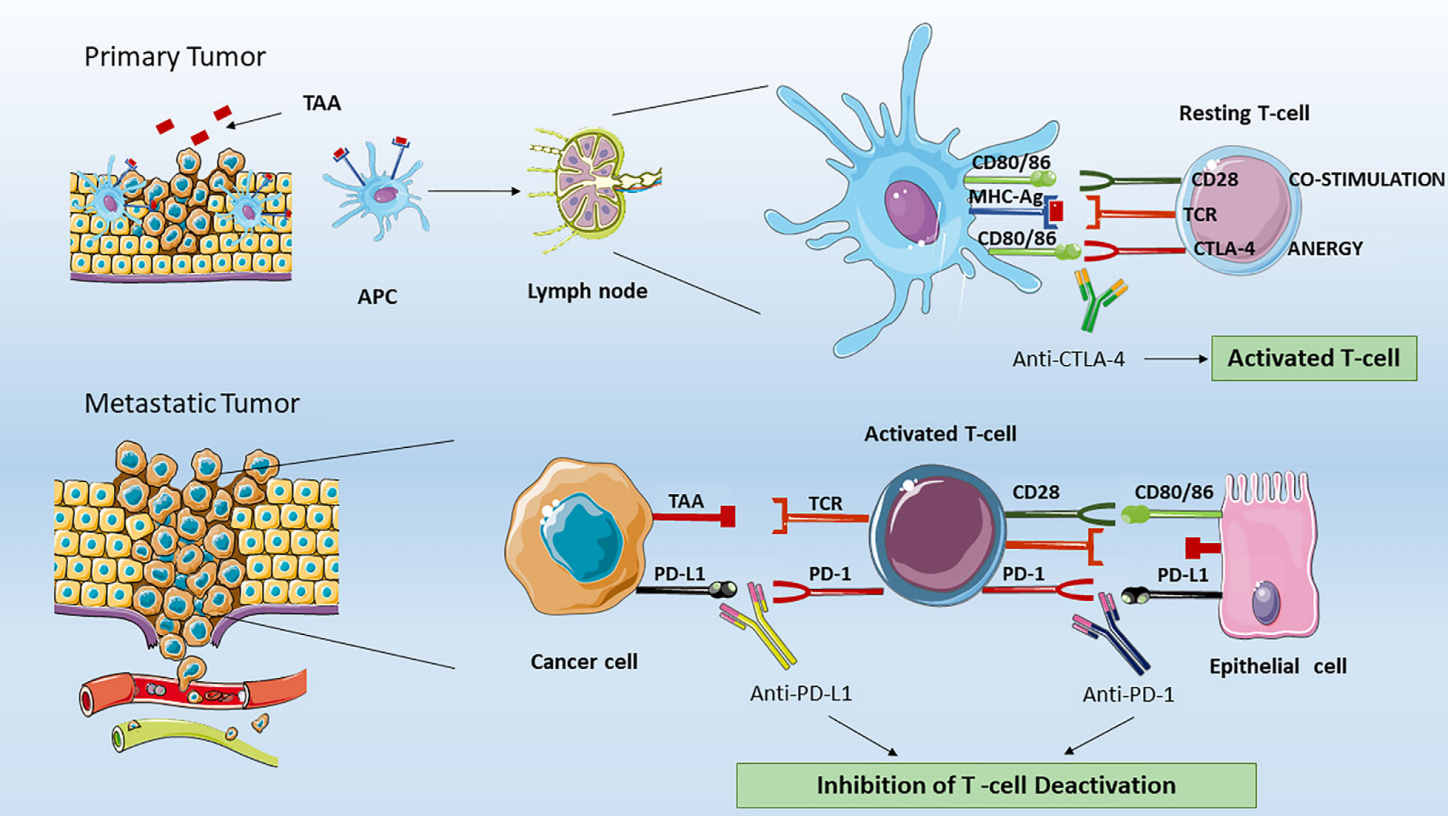
Effect of ICIs on T lymphocytes. In the tumor microenvironment, professional APCs, such as dendritic cells processed specific tumor peptides (TAA) and complexed them to MHC molecules. Then, APC migrated to T cell-dependent areas of tumor draining lymph node and presented TAA to naïve or quiescent T cells. In the immune synapse between resting T cell and APC, the lymphocytes activation is dependent by two signals. The first is mediated by the binding of TAA to T-cell receptor (TCR). The second signal could be activatory in the case of binding of T cell-CD28 to co-stimulatory CD80/CD86 or inhibitory. The latter is mediated by the binding of T cell-CTLA-4 to the same CD80/CD86 APC molecules. Therefore, CTLA-4 and CD28 compete for the binding to CD80/CD86 proteins. The CTLA-4 signaling will lead to T cell anergy by inhibiting the T naïve activation and clonal expansion. The anti-CTLA-4 blocking by monoclonal antibodies as ipilimumab restore CD28 pro-activatory signaling and result in effective anti-tumor T lymphocyte responses. In peripheral tissues, the activated T cell can be de-activated by the binding of PD-L1 (or PD-L2, not shown) expressed on tumor cells, organ cells or other immune cells to effector T cell- PD-1 receptor. The anti-PD-1 or anti-PD-L1 blocking by monoclonal antibodies (as Nivolumab, Pembrolizumab for PD-1 or Atezolizumab for PD-L1) ipilimumab restore CD28 pro-activatory signaling and restore effective anti-tumor T lymphocyte responses. APC, Antigen Presenting Cells; MHC-Ag, Major Histocompatibility Complex with tumor antigen; CTLA-4, Cytotoxic T-Lymphocyte Antigen 4; PD-1, Programmed cell death protein 1; PD-L1, Programmed death-ligand 1.

In the tumor microenvironment, cancer cells can evade the immunosurveillance by changing their surface antigens, thus avoiding the detection and destruction by host lymphocytes. A central mechanism of tumor-induced immune suppression is the increased expression of ligands able to bind inhibitory T cell receptors (2, 3, 5). These ligands are known as immune checkpoints and act in physiological conditions to prevent the development of autoimmunity at multiple steps during the immunological response. The main mechanisms involved in the T cell modulation are the suppression of potential autoreactive naïve- T cell (characterized by a TCR directed against self-antigens) at initial stages in lymph nodes, or in later phases the T cell deactivation in peripheral tissues (**Figure 1**). This process is called peripheral tolerance and is exerted mainly by the immune checkpoints cytotoxic T-lymphocyte–associated antigen 4 (CTLA-4) and programmed death 1 (PD-1) pathways. Tumor cells have developed ways to take advantage of peripheral tolerance by inducing a deranged immune checkpoint expression by T cell in order to avoid immune recognition (7, 8).

ICIs represent different classes of monoclonal antibodies that interrupt the delivering of inhibitory signals to T cells, and reprogram adaptive immunity to participate to cancer elimination.

Given that our immune system is ontologically programmed to recognize and eliminate what could ultimately harm our organism, an effective anticancer immune response is achieved through the interaction between the T-cell receptor (TCR) on quiescent T cells, and a tumor associated antigen (TAA) presented by antigen-presenting cells (APCs), mainly but not exclusively represented by dendritic cells, within molecules of the major histocompatibility complex (MHC). In the immune synapse, antigen binding to TCR, and the following T cell activation is functionally dependent on a second signal mediated by the binding of the T cell CD28 transmembrane protein, and APC CD80/86 (also indicated as B7-1/B7-2 ligands). The resulting intracellular pathway mediated by the non-variable CD3 coreceptor, culminates in T cell proliferation, differentiation, and cytokines (e.g. IL-2) secretion. Importantly, the absence of this co-stimulation leads to T cell impaired activation and apoptosis. To prevent an overstimulation, after antigen binding to TCR, the immune checkpoint protein CTLA-4 is shuttled from intracellular vesicles to T cell surface, where it exerts a co-inhibitory signal by competitively binding the same CD80/86 molecules on APCs (9). Since the lack of CD28-mediated second signal in presence of CTLA-4 results in T cell anergy, the inhibition of CTLA-4 receptor (by means of the use of specific monoclonal antibodies) allows T cell activation, thus restoring anti-tumor immunity.

CTLA-4 signaling occurs in the tumor draining lymph nodes (**Figure 1**). This signaling is initiated when an APC migrates from cancer peripheral tissues to T cell-dependent areas and presents a tumor-associated antigen (TAA) to a naïve T -cell. Interestingly, in contrast with CD28, which is constitutively expressed on naïve T cells, CTLA-4 appears to be induced after 48 to 72 h following TCR triggering, and has been showed to replace the CD28 signaling with higher affinity at a lower surface density. The CTLA-4 (CD152) is a type 1 transmembrane glycoprotein belonging to the Ig superfamily. Initially discovered in 1987 by Brunet JF et al. (10), during the screening of a mouse T-cell derived cDNA libraries CTLA-4 has been demonstrated to be expressed not only on activated T cells, but also on regulatory T-cells (Tregs), due to their high levels of FoxP3, which is known to regulate CTLA-4 expression (11). Regarding the signals transduced upon binding, the CTLA-4 cytoplasmic tail has been demonstrated to contain PI3K-like motif therefore suggesting an interaction with PI3K, MAPK, and NF-kB pathways.

In addition, beside the structural similarity with CD28, CTLA-4 receptors are capable to sequester CD80/86 from the surface of the APCs, resulting in significant depletion of the ligands on their surface. The role of CTLA-4 as essential “brake” on T cells to restrain immune responses was supported by studies performed in CTLA-4–deficient mice. The latter showed early after birth the development of lymphoproliferative disease, an impressive enlargement of lymphoid organs, and a lethal autoimmune phenotype (12, 13).

The hypothesis that CTLA-4 blockade could improve anti-tumor immune response was confirmed by Allison JP et al. in transplantable murine colon carcinoma, and fibrosarcoma, models (14).

A large body of experimental evidences confirmed the beneficial role of CTLA-4 inhibition in increasing immune recognition and elimination also of poorly immunogenic murine melanoma (15) and prostate cancers (16).

The CTLA-4 immune checkpoint provided the first target for the treatment of advanced melanoma. From initial murine studies and clinical trials, it took 15 years before the US Food and Drug Administration (FDA) approved ipilimumab, the first Ig1 human immunoglobulin monoclonal antibody directed against CTLA-4 (**Table 1**).

**Table 1:** Overview of principal ICIs, targeted tumor and clinical trials.

Immune checkpointed inhibited	Drugs	Year of approval	FDA-approved indications	Clinical Trial	
**CTLA-4**	**Ipilimumab**	**2011**	Metastatic melanoma	Non-smal cell lung carcinoma NCT03469960, NCT03351361, NCT02785952, NCT03302234	
** **			Renal cell carcinoma	Mesothelioma NCT02899299	
** **			Colonrectal cancer	Gastric cancer NCT02872116	
** **				Squamous cell lung carcinoma NCT02785952	
**PD-1**	**Nivolumab**	**2015**	Metastatic melanoma	Mesothelioma NCT03063450	
** **			Colonrectal cancer	Non-Hodgkin lymphoma NCT03366272	
** **			Classical Hodgkin’s lymphom	Metastatic clear cell renal carcinoma NCT01668784	
** **			Renal cell carcinoma	Head and neck cancer NCT02741570, NCT03342352	
** **			Non-small cell lung carcinoma	Lung cancer NCT03348904	
** **			Head and neck squamous cell carcinoma (HNSCC)		
** **			Metastatic urothelial carcinoma		
** **			Hepatocellular carcinoma (HCC)		
** **			Colorectal cancer with MSI-H		
** **	**Pembrolizumab**	**2015**	Metastatic melanoma	Small cell lung cancer NCT03066778	
** **			metastatic NSCLC	Renal cell carcinoma NCT03142334, NCT02853331	
** **			classical Hodgkin’s lymphoma,	Gastric adenocarcinoma NCT02370498	
** **			primary mediastinal B-cell lymphoma (PMBCL)	Urothelial carcinoma NCT02853305, NCT03244384, NCT02256436, NCT03374488,	
** **			Head and neck squamous cell carcinoma (HNSCC)	Colorectal cancer NCT02563002	
** **			gastric cancer	Pleural mesothelioma NCT02991482	
** **			solid tumors with MSI-H and MMR aberrations	Esophageal neoplasms NCT03189719, NCT02564263	
** **			metastatic urothelial carcinoma	Multiple myeloma NCT02579863, NCT02576977	
** **			Merkel cell carcinoma	Hodgkin lymphoma NCT02684292	
** **			renal cell carcinoma	Hepatocellular carcinoma NCT02702401, NCT03062358	
** **			Cervical cancer		
** **			Hepatocellular carcinoma		
** **	**Cemiplimab**	**2018**	Metastatic cutaneous squamous cell carcinoma	Cutaneous squamous cell carcinoma NCT04154943	
**PD-L1**	**Atezolizumab**	**2016**	Metastatic urothelial carcinoma	Renal cell cancer NCT02684006	
** **	** **	** **	Metastatic Non-small cell lung carcinoma	Gastric and gastroesophageal junction cancer NCT02625623, NCT02625610	
** **	** **	** **	Metastatic Small cell lung carcinoma	Ovarian cancer, fallopian tube cancer NCT03038100, NCT02839707, NCT02891824	
** **	** **	** **	Metastatic triple negative breast cancer		
** **	**Avelumab**	**2017**	Merkel cell carcinoma	Non-small cell lung carcinoma NCT02576574, NCT02395172	
** **	** **	** **	Metastatic urothelial carcinoma	Urothelial cancer NCT02603432	
** **	** **	** **		Diffuse large B-cell lymphoma NCT02951156	
** **	**Durvalumab**	**2018**	Metastatic urothelial carcinoma,	Non-small cell lung carcinoma NCT02273375, NCT02542293, NCT03164616, NCT02125461	
** **			Unresectable stage III Non-small cell lung carcinoma	Squamous cell lung carcinoma NCT02154490, NCT02551159	
** **				Urothelial cancer NCT02516241	
** **				Advanced solid malignancies NCT03084471	
**Combination of CTLA-4 and PD-1**	**Ipilimumab plus nivolumab**	**2016**	Metastatic melanoma	Non-small cell lung cancer NCT02659059	
		**2018**	Metastatic renal cell carcinoma	Metastatic renal cell carcinoma NCT0223174	
		**2018**	Colorectal cancer with MSI-H	Colorectal cancer with MSI-H NCT02060188	
		**2020**	Hepatocellular carcinoma (HCC)	Hepatocellular carcinoma (HCC) NCT01658878	

## PD-1 and PD-L1

At tissue level and in tumor microenvironment, cancer cells immune escape is mediated by the PD-1 inhibitory signaling (**Figure 1**). Normally, the PD-1 receptor (PDCD1 or CD279) is expressed on effector T cells, B and NK cells, while its ligands PD-L1 and PD-L2 are expressed in various types of self-cells (as tubular epithelial, endothelial cells, fibroblastic reticular cells, pancreatic islet cells, astrocytes, neurons) thus avoiding autoimmunity and host organ injury. PD-L2 expression is limited primarily to APC (17). More importantly, on T cells, the PD-1 expression is a feature of “exhausted” lymphocytes that have previously experienced high levels of stimulation. This state of exhaustion is frequently observed during chronic infections and cancer and is characterized by deterioration of T cell function, resulting in inefficient control of infections and tumors (18). On the other hand, cancer cells strongly upregulate PD-L1 ligands, and in metastatic tissues the PD-1 pathway on memory T cell causes T cell deactivation. PD-L1 increased expression has been assessed on cell surface in several types of cancers including melanoma, bladder, lung, kidney, colon, ovary, breast, glioblastoma, multiple myeloma and T-cell lymphoma. The main mechanism associated to enhanced PD-L1 expression on tumor cells have been correlated to PTEN deletion (19), PI3K signaling and persistent high IFNγ levels in the tumor microenvironment (20). Blocking the PD-1, PD-L1, and PD-L2 signaling by monoclonal antibodies allows tumor-infiltrating lymphocytes to be reactivated to identify and destroy malignant cells. Initially discovered from Ihshida Y et al. (21) as an immunoglobulin expressed on dying thymocytes, PD-1 would have been later associated as essential negative regulator of T cell response. In accordance, PD-1–deficient mice were showed to develop autoimmune disorders such as lupus like syndrome, characterized by glomerulonephritis and arthritis, and autoimmune cardiomyopathy (22). The binding of PD-1 is known to induce the phosphorylation of the tyrosine residue located within Immunoreceptor Tyrosin-based Switch Motifs (ITSM) of the cytoplasmic tails, leading to recruitment of phosphatases SHP1 and SHP2, and dephosphorylation of downstream effectors such as Syk, PI3K, and CD3 (17). Currently, several monoclonal anti-antibodies have been approved by the US FDA targeting PD-1 (i.e., pembrolizumab, nivolumab, and cemiplimab) and the ligand PD-L1 (atezolizumab, avelumab, and durvalumab) for the treatment of a number of different malignancies, including NSCLC, metastatic melanoma, bladder cancer, advanced renal cell carcinoma, and others (**Table 1**).

In summary, one CTLA-4 inhibitor and five PD-1/PD-L1 inhibitors have been approved by the FDA and others are undergoing testing within phase 3 clinical trials.

## Novel Target of ICIs

Apart from CTLA4-4 and the PD-1/PD-L1, novel checkpoints have been discovered, which can be targeted by specific monoclonal antibodies (23). Indeed, ongoing research is focused to the improvement of the clinical management of cancer patients treated with ICIs, in order to reduce the occurrence of immune adverse effect (including nephrotoxicity), and overcome the resistance after prolonged treatments (24). Several experiments led to hypothesize that the blockade of a single immune checkpoint may result into a compensatory enhancement of other checkpoint receptors in the tumor microenvironment (25). For that reason, research moved towards the synergistic effect obtained by the combined blockade of different immune checkpoints, as in the case of the combination of ipilimumab plus nivolumab (**Table 1**).

The next generation of immune checkpoints includes the lymphocyte activation gene-3 (LAG-3), T cell immunoglobulin and mucin-domain containing-3 (TIM-3), B and T cell lymphocyte attenuator (BTLA), T cell immunoglobulin and ITIM domain (TIGIT), V-domain Ig suppressor of T cell activation (VISTA), and B7 homolog 3 protein (B7-H3) (26).

LAG-3 (CD223) was first discovered by Triebel F et al. in 1990 as a novel lymphocyte activation gene closely related to CD4 (27). Further analysis of amino acid sequence would have revealed an approximately 20% of identity to CD4. LAG3 is expressed on CD4+ and CD8+ T cells, Tregs, B cells and plasmacytoid dendritic cells.

The LAG-3 signaling plays a negative regulatory role in T helper 1 (Th1) cell activation, proliferation, and cytokine secretion. Even if, given the high structural similarity between LAG-3 and CD4 should support the predominant binding to MHC-II, other molecules can interact with LAG3 as galectin-3 (28) , LSECtin , and α-synuclein (29).

However, the binding affinity of LAG-3 for MHC-II is 100-fold higher than CD4, thus MHC-II is considered the canonical ligand (26).

In a murine model of ovarian cancer, Huang R-Y et al. (25) explored the effect of combined blockade of LAG-3 and PD-1 pathways. Authors showed that the dual blocking suppressed tumor growth by enhancing CD8+ tumor infiltrating T cells and decreasing Tregs in the tumor microenvironment in synergic manner (26).

Furthermore, Huang R-Y et al. also supported the hypothesis of a compensatory mechanism. Indeed, when they evaluated the level of other inhibitory receptors, they found that in mice treated with anti-PD-1, the levels of LAG-3 and CTLA-4 were increased. In accordance, the anti-LAG-3 administration led to augmented PD-1 levels (25).

The results from Fourcade J et al. (30) and Koyama S et al. (31) provided similar insights also in melanoma and lung cancer.

Besides experimental model, the first single center, phase I trial was run in 2006 in stage IV Renal Cell Carcinoma patients (NCT00351949). The trial tested the monoclonal antibody anti-LAG3 named IMP321 (Eftilagimod alpha), initially proposed as a vaccine adjuvant (32). The trial showed an overall reduction of tumor progression, as well as increased levels of activated CD8+ T cells.

The combination with anti LAG3 and PD-1 has been assessed also in in patients with previously unresectable or metastatic NSCLC and with metastatic squamous cell carcinoma of the head and neck (NCT03625323).

A great repertoire of LAG-3 monoclonal antibodies and blocking agents is under active evaluation within ongoing clinical trials, making compelling results on safety, efficacy and potential nephrotoxicity, not yet available.

More than 20 clinical trials have been registered using the first commercially available monoclonal antibody directed against LAG-3 named Relatlimab (BMS-986016) (33).

In advanced solid tumor (as NSCLC, renal cell carcinoma, bladder cancer, squamous cell carcinoma of the head and neck and melanoma) the trial NCT01968109 is evaluating the efficacy of Relatlimab as a monotherapy or in combination with Nivolumab (an anti-PD-1 antibody).

## Immune-Related Adverse Events (irAEs)

In the past few years, treatment with ICIs dramatically improved the outcome of a number of solid tumors, extending progression-free and/or overall survival in patients with melanoma, NSCLC, urothelial cancer, renal cell cancer, and many other malignancies (**Table 1**). However, the exuberant activation of immune response generated by treatment with ICIs is complicated by a new class of side effects called immune-related adverse events (irAEs). IrAEs are often serious, characterized by clinical manifestations that closely resemble autoimmune diseases. Almost all organ and system can be affected by irAEs, mainly skin, gastrointestinal tract and liver followed by lungs, nervous system, endocrine organs, joints, heart, pancreas and the kidneys (34). Thus, the most frequent ICIs-induced irAEs are dermatitis, rash, vitiligo, colitis, pneumonitis, hypophysitis, hypothyrodims, and other endocrinopathies (35). The incidence of irAEs is wide, ranging from 15% to 90%, with severe forms ranging from 0.5% to 13% (36).

The manifestations occurred can vary depending on the type of ICIs used, although the frequency and severity are higher with anti-CTLA4 antibodies (especially ipilimumab) (37).

Even more severe (grade III and IV) toxicities may occur in as many as 20% of the patients treated with combined anti–CTLA-4 and anti–PD-1 agents. The timing of the onset of these irAEs varies widely, but appears to be within weeks to months of exposure, and may occur even after ICIs discontinuation (38). In addition, those irAEs that develop with one class ICIs (i.e. anti–CTLA-4) may not necessarily occur with exposure to another class (i.e. anti–PD-1/PD-L1) (34).

An emerging complication of ICIs administration is kidney damage, which includes acute kidney injury (AKI) – possibly evolving towards chronic kidney disease (CKD), proteinuria, and electrolyte abnormalities (5).

Originally, in contrast to extrarenal irAEs, the incidence of adverse effects affecting the kidneys appeared to be less common. Yet, epidemiological data were mainly retrieved by sparse, small case report, and were far from being reliable. In addition, too often oncologist reports these events just as “creatinine increase,” without further specifications. The estimated incidence of ICIs-associated AKI (ICIs-AKI) derives from Cortazar FB et al., who used pooled data from all phase 2 and 3 clinical trials published between 2014 and 2015, which enrolled at least 100 patients treated with ICIs (38, 39).

From a total of 3695 patients treated with ICIs monotherapy, overall incidence of AKI was of 2,2%. Regarding severe AKI, defined as an increase in serum creatinine (SCr) more than threefold above baseline, an increase in SCr to 4.0 mg/dl, or the need for renal replacement therapy (RRT), the detected incidence was lower (0.6%) (38, 39).

However, even if incidence of nephrotoxicity with monotherapy with any of the classes of ICIs was moderate, combinations including both an anti-CTLA-4, as well as an anti–PD-1, agent has been shown to be up to 5%. In particular, AKI was more common with combination therapy with ipilimumab/nivolumab combination therapy (4.9%) than with monotherapy with ipilimumab (2%), nivolumab (1.9%), or pembrolizumab (1.4%) alone (5, 35).

Fittingly, a meta-analysis by Manohara S et al., which evaluated 48 clinical trials that included 11,482 patients, reported an estimated incidence of ICIs-AKI of 2% (40). Consistently, Seethapathy H et al. (41) examined the incidence of ICIs-AKI in a setting of 1843 patients treated from May 2011 to December 2016 at the Massachusetts General Hospital. As estimated by the above mentioned meta-analysis, an incidence of 3% was determined. Interestingly, given the increasing use of these agents in a broad spectrum of malignancies (42), the incidence of relatively new irAEs such as AKI has been theorized to be rising from 9,9 to 29% in a near future (43).

## Clinical Features of ICIs-AKI

Previous case reports based the diagnosis of ICIs-induced AKI solely on renal biopsy (39, 44). The major histological features observed were acute tubular interstitial nephritis (ATIN) associated with edema, interstitial inflammation, and infiltration of T-lymphocytes, eosinophils and plasma cells. Urine analysis often displayed sterile pyuria and white blood cell casts (45, 46). In the last years, given the increased use of ICIs in a wide range of cancer, a need to harmonize the definition of AKI has emerged.

The definition and stage of AKI is regulated by the Kidney Disease Improving Global Outcomes (KDIGO) criteria according to relative changes in SCr (47). For instance, AKI stage II is defined as doubling of SCr, while stage III as tripling of SCr, or the need for RRT.

In order to standardize the ICIs-AKI definition across different studies, National Cancer Institute's Common Terminology Criteria for Adverse Events (NCI-CTCAEs) (48) describes AKI, in part, by comparing changes in SCr to the “upper limit of normal” cut-off parameters. In particular, the NCI-CTCAEs recognize five different grades of renal injury based on creatinine levels (grades 1–3), dialysis requirement (grade 4), and death (grade 5).

However, patients with cancer often have decreased muscle mass and these definitions may therefore be inadequate to detect increases in SCr that would fall within the “normal range”. Therefore, the application of NCI-CTCAEs criteria seem to fail in capture the lower grade kidney complications, and to completely ignore several AKI episodes that would have encountered as ICIs complication (5). These observations could explain the difficulty in the precise estimation of AKI in patient treated with ICIs. Additionally, for all definitions of ICIs-AKI, it is critical that the renal injury be directly attributable to the ICIs, and not to an alternative causes.

Another confounding element is the longer latency period between ICIs initiation and AKI development. In contrast with typical drug-induced acute interstitial nephritis (AIN) and to other extrarenal irAEs, the recently evaluated median time from ICIs initiation to AKI occurrence is 14 weeks (49), with several patients developing AKI later (6). As examples, an extrarenal irAE as dermatitis usually occurs within 4 weeks of treatment (50), whereas colitis within 6 weeks from ICIs start (49).

The delayed onset of AKI could be explained by the prolonged longevity of activated T-cells, rather than a direct toxicity of ICIs. Yet, even though all the ICIs have a long half-life of 2 to 3 weeks which allows longer intervals between dosing, the onset of AKI can occur from 8 months to 2 years (51) after treatment start. Thus, could not merely associated to classical drug nephrotoxicity as conventional chemotherapeutics. Furthermore, the pharmacokinetic of ICIs revealed that these drugs are not cleared by the kidney, but are primarily cleaved by proteolytic degradation within the target tissues by lysosomes, after receptor mediated endocytosis (5). For that reason, ICIs do not need dose adjustment for kidney impairment, and has been safely used in patients with end-stage renal disease (ESRD) (52, 53). However, patients with advanced CKD showed an increased risk of ICIs-induced AKI, therefore it is highly recommended that these patients should have a careful evaluation of renal function during treatment with ICIs.

As anticipated, a large body of evidences demonstrated that ATIN represents the most common histological finding in biopsies from patients experiencing ICIs-AKI (39, 45, 54–56).

However, the AIN induced by ICIs is closer to what observed during autoimmune diseases (46), as compared to drug hypersensitivity reactions (57). In a multicentric study that enrolled 138 patients with ICIs-induced AKI, ATIN was found in 93% of biopsied patients (6). This finding confirmed previous results in a series of 13 patients with ICIs-AKI in which the dominant pathologic lesion (observed in 12 patients) was AIN, characterized by diffuse interstitial infiltrates of CD3+ and CD4+ T lymphocytes and associated to granulomas in 3 cases (39). Thrombotic microangiopathy was a defining feature of one patient's pathology in this series, whose comorbid conditions included pre-existing hypertension.

In another smaller series, AIN was found in all 6 patients treated with ICIs who underwent kidney biopsy for AKI (54).

Glomerular lesions are less common, as compared to ATIN; however, in some case reports they have been associated predominately to the use of the anti-PD1 nivolumab. In one study of 16 patients with biopsy-proven ICIs-AKI, ATIN was present in 14 of the 16 cases, but co-occurred with glomerular disease in nine cases, including glomerulonephritis, IgA nephropathy, pauci-immune glomerulonephritis, and thrombotic microangiopathy (45).

Other small series have reported nephrotic syndrome with minimal change disease associated to the treatment with both anti-CTLA-4 (55), and anti–PD-1, antibodies (45, 58).

In a case report, Daanen RA et al. (59) described the occurrence of a severe nephrotic syndrome with AKI secondary to treatment with nivolumab in a patient with papillary renal cell carcinoma. Interestingly, during 8 weeks of nivolumab treatment, the patient showed AKI, hypoalbuminemia and proteinuria, whereas renal biopsy exhibited focal segmental glomerulosclerosis. In another case, Jung K et al. (60) presented the autoimmune glomerulonephritis as well as the tubulointerstitial injury in a patient treated with nivolumab for clear cell carcinoma. Interestingly, an immune complex-mediated glomerulonephritis with cellular crescents and necrosis was observed together with diffuse mesangial deposition of IgA, C3, and kappa and lambda light chains. At electron microscopy, one glomerulus showed several hump-like subepithelial deposits, and no subendothelial deposits and partial podocyte foot process abnormalities. Proximal tubules were flattened with simplified tubular epithelium and shorter microvilli. Pathologic examinations confirmed the final diagnosis of acute toxic-type tubular injury and IgA-dominant acute post-infectious glomerulonephritis (60).

These observations emphasize the heterogeneity of histopathologic features of injury from ICIs, as well as the immune activation seen in patients with ICIs-AKI. A further mechanism leading to podocyte foot process impairment, as observed for minimal-change disease and focal segmental glomerulosclerosis, could be the persistent, chronic release of inflammatory cytokines by T cells associated to ICIs-induced abrogation of CTLA-4 signaling (61).

Recently, risk factors for the development of ICIs-AKI have been evaluated (6). In the same multicenter study of 138 patients with ICIs-AKI, which included 276 unmatched control patients who received ICIs contemporaneously but did not develop AKI, Cortazar FB et al. identified three independent risk factors for ICIs-AKI: concomitant use of proton pump inhibitors (PPI), the combined treatment with anti–CTLA-4 and anti–PD-1/PD-L1 agents, and a lower baseline eGFR (6, 39, 41). Regarding the use of PPI as risk factor for ICIs-induced AKI, Cortazar FB et al. confirmed previous studies (62) describing an increased susceptibility to ATIN by the PPI in the general population. This event could be explained by the mechanism of reactivation of drug-specific T cell that will be further described. The finding that combination therapy is also associated to ICIs-AKI could be explained by the well-documented enhanced predisposition to irAEs.

The synergistic effect of dual checkpoint blockade was investigated by several researchers (63) mainly in murine model of advanced melanoma. Under the combined inhibition of the inhibitory receptors CTLA-4 and PD-1, tumor-infiltrating T cell numbers increased, a change in the ratio of effector T cell to Tregs was induced, and effector T cell function was improved. Given these promising animal models, Wolchock et al. (64) tested the combination of ipilimumab and nivolumab in metastatic melanoma patients. Although objective clinical responses were found in the range of 40%, more than 53% of patients exhibited grade 3 and 4 toxicities. Combination therapy with nivolumab plus ipilimumab has resulted in a prolonged overall survival also in patients with renal cell carcinoma (65). Nevertheless, the study from Motzer RJ et al. showed an incidence of any-grade irAEs of 93%, despite an objective response rate of 42%.

Finally, in a recent open-label, phase 3 trial involving patients with advanced NSCLC, treatment with nivolumab plus ipilimumab resulted in a longer duration of overall survival (66), while the percentage of patients with grade 3 or 4 treatment-related adverse events was 32.8%.

These results clearly demonstrate the superiority of the combination of ipilimumab and nivolumab over monotherapy, and the heterogeneity in irAEs depending on cancer type. To date, grade 3 and 4 irAEs are frequent, although these effects are usually easily manageable, at least in referral centers where ICIs are commonly administered.

## Mechanisms of ICIs-Induced AKI

**Figure 2:**
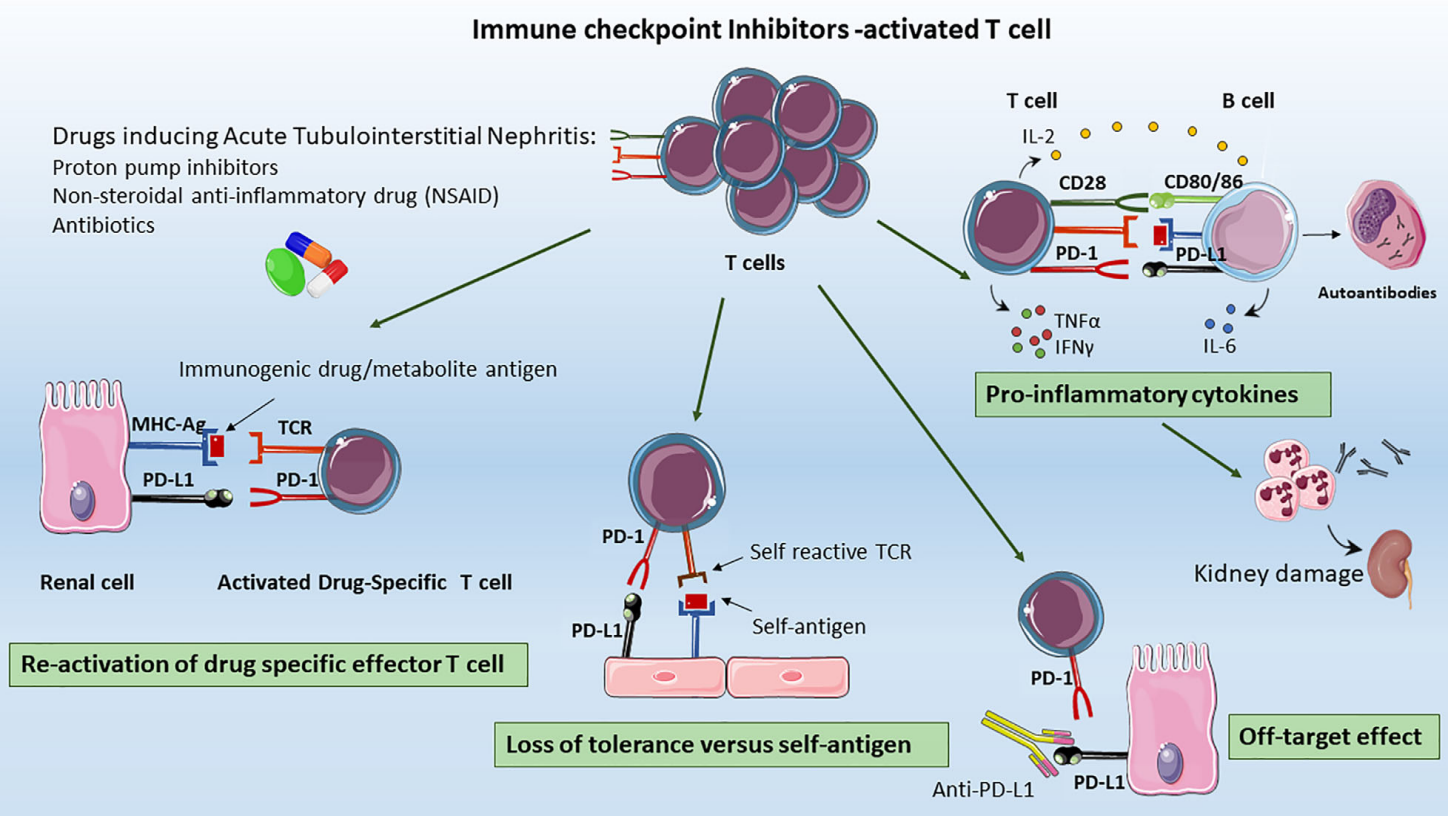
Mechanisms of ICIs-associated AKI. The proposed mechanisms underlying ICIs-induced AKI include: *Re-activation of drug specific T cells:* T cell primed by different drugs (e.g. previous or concomitant antibiotics, PPIs, or NSAIDs) became latent over the time; however they can be re-activated by ICIs, leading to loss of tolerance*; Loss of tolerance versus self-antigens:* the formation, the selection and proliferation of a clone of self-reactive T-cells, the auto-reactive T cell could activated self-reactive B cells leading to auto-antibody release, that to renal injury; *Off Target Effect*: the upregulation of PD-L1 on renal tubular epithelial cells can lead to kidney damage by effector T lymphocytes infiltration resulting in acute tubulointerstitial nephritis, *Pro-inflammatory cytokines:* ICIs promote the migration and activation of effector T cells in renal tissue, the infiltration of other immune cells as B cells together with pro-inflammatory cytokines release as CXCL10, TNFα, IL-6 that contribute to the generation of an inflammatory milieu, leading to renal damage.

Although the mechanisms underlying ICIs-induced AKI are yet to be elucidated, some hypotheses have nevertheless been advanced, based on murine models and commonly observed extrarenal irAEs (**Figure 2**).

First, CTLA-4 and PD-1 inhibition could lead to the development of autoantibodies against self-antigens present on tubular epithelial cells, mesangial cells, or podocytes (56). Relevantly, ipilimumab treatment was associated to a lupus-like glomerulopathy, and to serum circulating levels of anti dsDNA and anti-nuclear antigen antibodies closely resembling the autoimmune lupus nephritis phenotype (4, 67). More importantly, the level of circulating autoantibodies appeared to be restrained by ICIs interruption, and glucocorticoid administration (56, 68) (**Figure 2**).

Second, another mechanism could be the development, the proliferation and the aberrant activation of a clone of self-reactive T-cells. This hypothesis can be supported by the presence of a robust infiltration of effector T-cell in organs not related to the tumor, which presented an impressive high level of similarities in TCR sequence. Intriguingly, Johnson DB et al. reported the cases of patients with melanoma treated with ipilimumab and nivolumab in whom fatal myocarditis developed. Within the tumors of these patients, Authors observed high levels of self-muscle-specific antigens (desmin and troponin) indicating that T cells could be targeting an antigen shared by the melanoma, skeletal muscle, and the heart (69).

It is reasonable to hypothesize that also an intrinsic kidney antigen, initially tolerated but recognized as non-self with the brake of CTLA-4/PD-1 signaling in self-reactive T cells could be responsible for acute tubulointerstitial nephritis (70, 71). It has been reported that some auto-reactive T cells escape negative selection in the thymus and are kept dormant by several mechanisms to prevent autoimmunity. Further studies are required to demonstrate the TCR clonality in tumor and kidney in ICIs-T cells-related nephrotoxicity.

An alternative hypothesis is that renal tubular cells express PD-L1, which protects them from T-cell-mediated autoimmunity. Ding H et al. showed that PD-L1 is constitutively expressed on HK-2 cells, and is dramatically upregulated by IFNγ. In normal kidneys, in situ hybridization and immunohistochemical staining revealed constitutive low expression of PD-L1 on proximal tubules at both mRNA and protein levels. However, PD-L1 higher expression was found in kidneys with type IV lupus nephritis. In vitro, pre-treatment of IFNγ-stimulated HK-2 cells with anti-PD-L1 significantly enhanced IL-2 secretion from co-cultured, mitogen-activated Jurkat or human peripheral blood T cells (72, 73). Therefore, anti-PD-L1 antibodies administrated for cancer immunotherapy could bind other sites than T cell or cancer cells leading to organ-specific injury (74, 75). However, given that ipilimumab is a fully human IgG1 characterized by the lack of antibody-dependent cell-mediated cytotoxicity and complement-dependent cytotoxicity, the underlying mechanisms of renal injury deserve more investigation (23). Together with PD-L1, renal allograft cells have been shown to upregulate also PD-1 during acute rejection as a protection mechanism of tubular cells from T cell mediated injury. The PD-1 increased level and the consequent enhanced PD-1/PD-L1 on Tregs has been extensively demonstrated to be beneficial during renal ischemia/reperfusion injury (IRI) (76, 77). In a mouse model of IRI, PD-L1 or PD-L2 blocking by monoclonal antibodies, reduced Treg-mediated protection and significantly exacerbated the loss of kidney function, renal inflammation, and acute tubular necrosis (76) (**Figure 2**).

Thirdly, another explanation for ICIs-induced AKI is the reactivation of drug specific T cell through ICIs loss of tolerance. In the majority of reports, patients received concomitant medications as PPI and nonsteroidal anti-inflammatory drug (NSAID) to treat ATIN. The ATIN-drug exposure, iatrogenic and xenobiotic molecules can trigger an immune response either by itself or after binding to tubular antigens, thus acting as haptens. These T cell primed by drugs administration became latent over the time, however by ICIs they can be reactivated leading to loss of tolerance. Accordingly, in a patient with NSCLC treated with the anti-PD-1 antibody nivolumab who developed kidney injury, circulating lymphocytes appeared to be effectively stimulated by the PPI lansoprazole (78). Strikingly, patient showed rapid improvement in kidney function in 3 days (creatinine decreased from 2.74 to 1.82 mg/dl) upon discontinuation of lansoprazole (78). In summary, patients with ICIs-induced AKI receiving a concomitant drug (e.g. NSAID) showed a greater probability to completely recover renal function after the interruption of the drug (6, 78). This effect may be explained by T cell reactivity to the drug rather than to endogenous autoantigens; therefore, the cessation of the offending NSAID, antibiotics, or PPI would lead to a more rapid attenuation of T cell immunologic activity (38).

Furthermore, in the first multicentric study of 138 patients evaluating the clinical feature of ICIs-AKI, (6) nearly 70% of the patients with ICIs-AKI were receiving an ATIN related medication. In particular, 9% was receiving antibiotics, 22% NSAID and, surprisingly, 54% PPI. The latter recently emerged as the most common causes of drug-induced ATIN. Therefore, PPI should be used with caution in patients receiving ICIs treatment, and should be discontinued in those who develop ICIs-AKI.

In brief, anti–PD-1 antibody treatment can disrupt the peripheral immune tolerance between renal tubular cells, dormant auto-reactive T-cells, and tolerogenic dendritic cells (79). The prevalence of immature and functional defective plasmacytoid dendritic cells could also explain the development of tubulointerstitial nephritis after PD-1 therapy, irrespective of whether re-activated T-cells recognize kidney intrinsic antigens or specific drugs.

Finally, treatment with ICIs promotes the migration and activation of effector T cells in renal tissue and the infiltration of other immune cells together with pro-inflammatory cytokines release. In accordance, patients treated with ICIs exhibit increased serum level of CXCL10, TNFα, IL-6 that contribute to the generation of an inflammatory milieu, leading to renal damage (74). The importance of the increased cytokines levels has recently emerged in association with cytokine release syndrome (CRS), a serious complication of the switch from immunotherapy to targeted therapies (80). There are limited data regarding the efficacy of treatments in ICIs‐AKI. Small case series have shown recovery of renal function with glucocorticoids in the majority of cases (39, 41, 54). A recent multicenter study from Cortazar FB et al. (6) demonstrated that the glucocorticoid treatment in a cohort of ICIs-AKI patients was independently associated with complete renal recovery. However, we are still far beyond the identification of a glucocorticoid regimen to prevent the progression of AKI.

## ICIs and Kidney Transplantation: The Switch to mTORi as a Strategy

Kidney transplantation is a life-saving therapy for patients with ESRD leading to improved survival and quality of life (81). Immunosuppression may increase susceptibility to cancer by inhibiting immune surveillance, predisposing to oncogenic viral infections, and reducing the rate of DNA repair (82). The risk of cancer is two to four-fold higher in transplant recipients as compared to age-, sex-, and race-matched individuals from similar geographic areas (82). This higher risk is often associated to prolonged immunosuppression that revert the balance between graft immune-tolerance and anti-tumoral immunity. After a solid transplant, the most common occurring cancers are the carcinomas of the skin, non-Hodgkin's lymphoma, Kaposi sarcoma, lung and cancer of the transplanted organ (i.e. liver or kidney) (83, 84). Besides the occurrence of the *de novo* cancer, patients with a history of cancer before transplantation are more likely to experience early cancer relapse, usually within 2 years after transplantation (85, 86).

The success of the first clinically approved ICIs has created an increased appreciation of immunotherapy also in transplanted patients.

Currently, there are no guidelines for the treatment of ICIs requiring transplanted patients since they have been excluded from all clinical trials of ICIs, and there are no randomized control trials. Conceptually, the crucial core of ICIs is whether stimulates the immune system to destroy the cancer cells, or else suppress immune response to prevent allograft rejection. In order to find a balance between ICIs-mediated T cell stimulation, and anti-rejection immunosuppression, case reports and small case series of transplanted recipients receiving ICIs have slowly appeared. By reviewing case reports, the frequency of rejection with the anti-CTLA-4 ipilimumab monotherapy emerged lower (33%) compared to patients treated with anti–PD-1 monotherapy (52%) (87).

The higher rate of rejection with anti–PD-1 treatment is not surprising. The PD-1/PD-L1 signaling has been described as pivotal in peripheral organ transplant homeostasis (88) and the PD-L1 overexpression in renal tubular cells is a mechanism to modulate T cell activation (72).

Regard the rejection type, acute rejection of transplanted kidney after PD-1 inhibitors occurs mainly through T cell mediated rejection, although antibody mediated rejection has been also observed (89).

Acute T-cell-mediated rejection after administration of PD-1 inhibitors may be explained by the ICIs-induced activation of T-cells against donor allograft antigens. This loss of tolerance leads to graft failure via T-cell infiltration in the renal interstitium, damaging renal tubular epithelial and endothelial cells. These findings are in line with the reported AIN, characterized by infiltration of T-cells and granulocytes in renal tissue, after treatment with ICIs in non-transplanted patients (90). As concerns the antibody-mediated rejection, it may be attributed to the proliferative response of B-cells induced by activated T-cells or activation of memory B-cells expressing PD-1 induced by the reduction in immunosuppressant use during PD-1 inhibitor treatment (91).

The sharp rates of rejection in transplant recipients under ICIs medications have led to the development of strategies before the initiation of ICIs. These approaches includes modification of dosage of immunosuppressive medications, the pre-emptive switch to corticosteroids and, more importantly, the switch from a calcineurin inhibitors (CNI) to mTOR inhibitors (mTORi) before ICIs initiation.

Lowering the dose of immunosuppressant has been considered a crucial management strategy in some cancers, such as post-transplant lymphoproliferative disorders and skin cancer. However, the reduction of immunosuppressive drugs before the initiation of PD-1 inhibitor has been observed to significantly increase the risk of graft failure. Indeed, it is well known that immunosuppressive therapies are vital in regulating acute allograft rejection and inducing long-term transplanted kidney survival (92–94).

In 1999, the main mTORi (sirolimus) obtained FDA approval for use in clinical kidney transplantation (95). Since then, an extensive literature has emerged not only on its effects on graft survival, reduced rejection and mortality but also on other important clinical outcomes, such as malignancy, cardiovascular disease and infection (84, 96). Thus, the majority of studies of mTOR inhibitors involved conversion from CNI either early (2–6 months) or late (>6 months) post-transplantation (97).

A large body of evidences suggest that early conversion from a CNI to an mTORi-based maintenance regimen can reduce the development of malignancies as non-melanoma skin cancer in transplant recipients (98–101).

The mTOR pathway is a key regulator of immune cells metabolism, proliferation and anti-inflammatory reactivity in both innate (dendritic cells and macrophages) and adaptive effectors (T and B lymphocytes) (102). As widely discussed, this pathways is often dysregulated in many types of solid and hematological malignancies (103). Therefore, in this scenario, mTORi are used both as immunosuppressive strategy to prevent graft rejection in transplanted patients and as antitumor therapy, indicating that a careful immunosuppressive dose modification in combination with immunotherapy should be administrated. However, to the best of our knowledge, in transplanted patients with *de novo* cancer, there is no consensus on immunosuppressive treatment schedule, since early phase clinical trials are still ongoing (CA209-933ISR).

In an elegant study Sabbatini et al., investigated the oscillatory inhibition of mTOR activity in kidney transplant recipients and found that lower level of everolimus were able to induce a robust proliferation of Treg by TCR triggering, a decrease of neutrophils and CD8 T cells and a reduced proinflammatory activity. Authors then hypothesized the possibility that management of mTORi dosage level (lower by six to 10-fold than in the oncology setting) and administration schedule (twice versus once a day in cancer therapy) could target respectively immune tolerance or cancer growth control (104, 105).

The switching to mTORi, together with the dose reduction of other immunosuppressive drugs has been associated to better overall survival of both oncologic patients and graft independently of the stage, type of carcinoma and oncologic treatment. Indeed, in a monocentric cohort of more than 500 kidney and liver allograft recipients with *de novo* cancer, Rousseau B et al, demonstrated that mTOR inhibitor introduction with optimal oncologic treatment significantly improved survival of patients (106). Vanasek TL et al. showed that treatment with mTOR inhibitors and concomitant ICIs could maintain T-cell anergy (107). In addition, mTORi have been demonstrated to stimulate naïve T-cell differentiation into Tregs, especially in the presence of IL-2 (108). Therefore, it is arguable to sustain that treatment with mTORi not only could reduce cancer progression in a broad spectrum of malignancies, but could exert anti-tumor effects (109).

In a case report, a kidney transplant recipient was treated with nivolumab for metastatic duodenal adenocarcinoma. Immunosuppressive regimens included concurrent prednisolone and the mTOR inhibitor sirolimus. Tacrolimus was replaced by sirolimus before anti–PD-1, and serum sirolimus levels were initially maintained at lower levels (4–6 ng/mL) after anti–PD-1, and then increased to regimen values (10–12 ng/mL) 2 weeks after. Intriguingly, the patient maintained renal graft function without tumor progression (110).

In addition, in a recent case report Esfahani K et al. (111) investigated the effect of the combination of mTORi and an ICIs (sirolimus plus pembrolizumab) in a kidney transplanted patient with melanoma. Interestingly, the ICIs-mTORi combination decreased the global CD8+ T cell activation responsible of ICIs-induced kidney allograft rejection. Furthermore, the dual therapy did not reduce the IFN-γ–producing CD4+ T cells that persisted in circulation. Thus, mTORi supported the immune tolerance while potentially adding anti-tumor efficacy to PD-1 blockade in patients with metastatic melanoma.

Finally, the ability of sirolimus to prevent T cells responses against renal allograft was investigated in 64 patients of multicenter trial (101).

Euvrard S et al. randomly assigned transplant recipients who were taking CNI and had cutaneous squamous-cell carcinoma either to receive sirolimus as a substitute for CNI or to maintain their initial treatment. Strikingly, switching from CNI to sirolimus led to longer disease-free survival among kidney-transplant recipients with previous squamous-cell carcinoma (101). Further studies are necessary to assess the potential effect of conversion from CNIs to mTOR inhibitors on both rejection and cancer. However, as the incidence of rejection in patients receiving ICIs therapy is very high, the general recommendation is to frequently monitor patients' kidney function by weekly SCr during all treatment.

## Biomarkers for ICI-Based Immunotherapy and ICIs-Induced AKI

ICIs-based immunotherapies has been shown to improve survival of patients in several types of advanced cancers (112). However, despite the promising results in terms of overall survival, many patients still experience severe irAEs (2, 36). There is a critical need to define biomarkers that can anticipate clinical outcome and the risk of organ toxicity in patients receiving ICIs. Major efforts in biomarker studies are ongoing and ICIs appeared encouraging (113). Several candidates have been proposed including the body composition parameters (e.g., age>75 years and female gender) (114, 115), systemic non-invasive biomarkers, tumor associated molecular features (e.g., PD-L1 expression and tumor mutation burden) (116, 117) and commensal bacteria (118). For the large part of these factors, the validation in independent patient cohorts with large sample size is still required (119). Nevertheless, some factors have been approved by FDA to select patients that would benefit from the treatment (e.g., PDL1 expression as a biomarker for patient selection) (120, 121). Despite promising results, more research is required to identify and validate the exact combination of biomarkers able to predict treatment outcomes and the occurrence of kidney nephrotoxicity (122). Here we will summarize the principal biomarkers associated to ICIs-irAEs and that could correlate with the development of AKI.

## Systemic Biomarkers of irAEs and ICI-Induced AKI

Systemic biomarkers of irAEs as lymphocytes and eosinophils counts, neutrophil-to-lymphocytes ratio (123, 124), and cytokine circulating level have been assessed due to poor invasive and routine measurements.

Regarding blood cell count, the higher number of eosinophils and T lymphocytes has been demonstrated to correlate with better survival in melanoma patients treated with pembrolizumab (125). Recently, Nakamura Y et al. and Diehl A et al. provided evidence that in melanoma and renal carcinoma eosinophils counts was also associated to the incidence of irAEs (124, 126).

In the plethora of pro-inflammatory cytokines, IL-17 is associated with autoimmune disease like rheumatoid arthritis, psoriasis, and inflammatory bowel disease (127) (i.e. Chron's disease). Therefore, the evaluation of IL-17 levels in ICIs-treated patients seems more than reasonable (128).

IL-17 is mainly released by Th17 CD4+ cells that are potent inducers of autoimmunity and are regulated by CTLA-4. Indeed, CTLA4 blocking by means of tremelimumab has been demonstrated to increase Th17 cells in peripheral blood of patients with metastatic melanoma and to correlate with autoimmune toxicity (129). Tarhini AA et al. reported that higher levels of circulating IL-17 at baseline associated with incidence of irAEs as diarrhea and colitis in melanoma patients treated with ipilimumab. In addition, TGF-β1 and IL-10 levels were associated with clinical outcome (128).

Besides irAEs, IL-17 is also over-released during AKI and associated with poor outcome (130). In a recent study, Maravitsa P et al. showed that IL-17 was the only cytokine highly produced from peripheral blood mononuclear cells (PBMCs) and CD4-lymphocytes of patients with septic shock and AKI, and that was gradually consumed from the kidney (131). Interestingly, a persistent increase in circulating Th17 cells was observed in mice model of renal IRI and correlated with systemic organ damage as pulmonary fibrosis (132). Similar results were found in renal transplanted patients with Delay Graft Function (130). Another promising biomarker of irAEs is CD163, a receptor expressed from M2 macrophages that are largely present in the tumor microenvironment (133). M2 macrophages are characterized by immunosuppressive properties, thus often associated with poor prognosis. The soluble sCD163 obtained by the proteolytic shedding of the receptors is increased in autoimmune disease (134) and fittingly, in melanoma patients during anti-PD1 treatment (135).

Recently, the conversion of pro-inflammatory (M1) to anti-inflammatory (M2) macrophage types has obtained a renewed appreciation particularly during the AKI-to-CKD transition (136).

In a cohort of sepsis patients, the diagnosis value of urine sCD163 levels were evaluated for predicting AKI occurrence, as well as for assessment of patients' prognosis (137). More recently, Sun PP et al. enrolled 205 patients with renal intrinsic AKI revealing a sharp augment of urinary sCD163 in glomerulopathy cases (138). In addition, urinary CD163 showed better diagnostic performance in differentiating disease etiologies compared to traditional urinary biomarkers of AKI (i.e. NGAL and KIM-1). Similar findings were reported in human ATIN biopsies (139). Kim M-G et al. showed a positive correlation between the density of CD68+ macrophages and the severity of AKI, whereas the density of CD163+ M2 macrophages was associated with a lack of renal functional recovery.

Inflammation has an integral role in the pathophysiology of irAEs. The principal pro-inflammatory cytokine IL-6 can promote tumor progression via inhibition of cancer cell apoptosis as well as promotion of angiogenesis. Plasma increased IL-6 levels have been correlated with poor overall survival in melanoma patients treated with ICIs-based immunotherapy (115).

In accordance, IL-6 is commonly elevated in inflammatory arthritis following ICIs therapy (140) as demonstrated in several type of cancer (e.g. malignant melanoma) (141).

From a renal perspective, IL-6 is a well-recognized biomarkers of renal injury (142, 143) and has been evaluated as central tool for predicting the development of AKI in critically ill patients (144, 145) as well as in the recent pandemic COVID-19 disease (146).

The measurement of the serum enzyme lactate dehydrogenase (LDH) is well recognized in the follow-up of patient with metastatic melanoma (147, 148) as it has prognostic value in renal cell carcinoma (149).

LDH is released by rapidly growing tumors characterized by a high cellular turnover. Recently, LDH has emerged as an independent factor for poor prognosis in patients with advanced melanoma (147, 150) treated with ipilimubab (151), nivolumab, and pembrolizumab (152). Similar results were reported also in NSCLC (153).

In the kidney, LDH has been shown to correlate with the principal parameters of kidney impairment (including estimated glomerular filtration rate (eGFR), microalbuminuria and proteinuria) (154, 155); in addition, urinary LDH has been evaluated in the early detection of acute tubular necrosis (156, 157) preceding AKI.

Altogether, these results lead us to speculate that IL-17, sCD163, IL-6, and LDH levels during ICIs treatment may serve as predictive markers for irAEs and evaluated in combination with other urinary markers of AKI (commonly KIM-1, L-FABP, IGFBP7, IL-18) could provide additional information also for the risk of ICIs-induced AKI.

## Immunosenescence and Cell Cycle Arrest as Biomarkers of ICIs-Induced AKI

Immunosenescence describes the process of progressive deterioration of the immune functions during aging due to a several causes such as: (i) reduced NK cell-mediated cytotoxicity and perforin release, (ii) altered TLRs and NODs activation on monocytes, decreased phagocytosis, ROS generation; increased basal production of proinflammatory cytokines; (iii) less efficient antigen presentation and phagocytosis by dendritic cells; reduced secretion of IFNγ and IL-12, (iv) systemic inflammaging as a state of chronic, low grade inflammation and the (v) thymic involution leading to reduced naïve T cell and increased memory cells in the elderly (158–160). Dysregulated functions associated to immunosenescence can include reduced responses to vaccination, lower antitumor ability of CD4, CD8 T cells and APC, increased systemic inflammation, as well as autoimmunity (79). Aging contributes to a reduced repertoire of naive CD8+ T cells and to an increased pull of memory, senescent or exhausted T cells, hence leading to a decline of adaptive immunity (161, 162).

Several clinical trials have observed the impact of immunosenescence on the effectiveness of ICIs. From a bird eye view, aged patients benefit less from the PD-1 inhibitors and CTLA-4 inhibitors in certain cancers, even though several exceptions have been reported (163).

A possible explanation behind this observation is that ICIs rely on intact immune responses to tumor neoantigens, thus a misbalance in immunocompetent T cells significantly impaired the efficacy of treatments.

However, even if meta analyses suggested the correlation between poor survival benefit for anti-PD-1 agents and age older than 75 years, it should be observed that chronological age does not necessarily reflect biological age of immune system. Besides age, many other conditions can induce immunosenescence since caloric restriction, nutrition or physical activity can delay this process (158) .

Recently, Moreira A et al. analyzed immunosenescence markers from PBMC of patients with newly diagnosed, untreated, metastatic melanoma (164). Regardless to patients' age, the Authors demonstrated that the loss of senescence markers on PBMC is correlated with clinical response to ICIs. These markers included CD27 and CD28, as well as Tim-3 and CD57 (165). The loss of CD27 and CD28 on CD4+ and CD8+ T cells, as well as the expression of the Tim-3 and CD57, all of them senescence markers, correlated with resistance to ICIs. In particular, the mucin domain containing protein T-cell immunoglobulin-3 (Tim-3) is a marker for T-cell exhaustion and combined PD-1/PDL1 and Tim-3 blockade have been proposed to prevent T-cell exhaustion in patients with hematologic malignancies (164).

Latterly, Zaretsky JM et al. performed a whole-exome sequencing in biopsy samples from metastatic melanoma patients treated with anti–PD-1 therapy (166), reporting that resistance to ICIs was associated with defects in the interferon pathway that plays an important role in immunotherapy resistance mechanism since it can induce cell senescence (167, 168). Thus, the disruption of INF-γ-induced cellular senescence could partially explain late acquired resistance to ICIs and disease progression.

In recent clinical trials, cell cycle arrest biomarkers as tissue inhibitor of metalloproteinase 2 (TIMP2) and insulin-like growth factor binding protein 7 (IGFBP7) have been demonstrated to be effective in the early detection of AKI (169–171), and to perform better than other biomarkers such as NGAL, IL-18 (172), L-FABP, and KIM-1 (173, 174). During AKI, in response to tubular injury or DNA damage, IGFBP7 is highly expressed and directly can increase the expression of p53 and p21, whereas TIMP2 promoted the augment in p27. The proteins p53, p21 and p27 together with p16 blocked the cyclin-dependent protein kinase (CDKs) od cell cycle resulting in G1 phase arrest (171). Furthermore, AKI is associated to progressive increased level of other cell cycle arrest markers as p16 and p21 and klotho reduction (175). The premature renal aging has been observed in several model of IRI-induced AKI with p21 augmented amount both at renal (176–178) and at urinary levels (179).

In conclusion, cell cycle arrest biomarkers could represent a step forward toward prediction of ICIs response and recognition of ICIs-induced AKI. Additional validation studies are needed in order to fully characterize their clinical usefulness in combination with other markers, in order to predict survival, occurrence of irAEs, and renal function deterioration (180).

## Gut Microbiome as Biomarkers of irAE and ICI-Induced AKI

The gut microbiome is composed by more than 3.8 × 10^13^ bacteria able to maintain host physiology and immune homeostasis (181). Recent advances in metagenomic analysis has improved our understanding of microbiota-related effects in health and disease. Alterations in intestinal microbiota dynamics (dysbiosis) has been linked to multiple human diseases, including intestinal disorders and cancers (182). In addition, gut microbiota composition has been associated to ICIs response, ICIs-induced irAEs (as colitis) and AKI (119).

Regarding response to ICIs, through the analysis of fecal samples and gut bacteria identification, several authors showed that bacteria composition correlated with immunotherapy response in the treatment of melanoma (183–185), renal cell carcinoma (118, 186) or NSCLC (118).

In melanoma patients, Chaput N et al. provided evidences that a microbiota enriched with *Faecalibacterium* genus and *Firmicute*, instead of *Bacteroides*, was associated to a better outcome during ipilimumab therapy (183).

Furthermore, Gopalakrishnan et al. by performing a bioinformatics analysis of gut microbiome samples of melanoma patients indicated that higher diversity and abundance of the *Ruminococcaceae* family bacteria was protective before anti-PD treatment (185). However, despite the study from Chaput N et al. and others (183, 185, 187) indicated a better outcome in *Faecalibacterium* and *Firmicute* gut microbioma, they also revealed a higher frequency of ICIs-induced irAEs such as colitis. In the plethora of commensal bacteria, Routy B et al. identified that the *Akkermansia muciniphilia*, one of the most abundant bacteria in the ileum microbiota, was able to strengthen the efficacy of anti-PD1 therapy by reinforcing intestinal barrier integrity and reducing systemic inflammation (118, 184).

Gut microbiota appears to have a central in the progression of renal injury since strongly linked to uremic toxins (188, 189). Several studies the improvement of CKD and ESRD after gut microbiota-directed intervention (190, 191). Bidirectional interaction between gut microbiota and kidney is being recognized as an important modulating factor in AKI (192).

The profile of AKI-microbiota has been recently characterized by metagenomic sequencing. Interestingly, the abundancy of *Erysipelotrichia*, *Lactobacillus* *salivarius* and *Bacteroides sp* in rodent model of renal IRI and cisplatin-induced AKI was reported (193, 194).

Other data supports the hypothesis that gut microbiota influence kidney function and kidney resident immune cells through Short Chain Fatty Acids (SCFAs). SCFAs (as acetate and propionate) are produced as the end products of the fermentation of dietary fibers by gut microbiota, and are released into the systemic circulation (193, 195). Administration of SCFAs was found to significantly improve renal dysfunction in a model of IRI-induced AKI (195).

It is well known that antibiotics can perturbate the gut microbiota and increase the risk of developing inflammatory bowel disease (196). Interestingly, the transfer of an antibiotic-perturbed microbiota from mouse mother to newborn promoted and accelerated the development of gut inflammation in the offspring (197).

Regarding the combination with antibiotics, Routy B et al. showed that dysbiosis generated by administration of antibiotics significantly affect antitumor response to ICIs in both mice and humans (118). Similar results were found by Jang HR et al. that demonstrated commensal microbes have a protective role in the pathogenesis of AKI, since they regulate CD8 T cells trafficking and modulated renal inflammation and injury (198). In contrast, depletion of gut microbiota using broad spectrum antibiotics protected from IRI-induced AKI by reducing maturation status of F4/80+ renal resident macrophages and bone marrow monocytes (199).

Finally, gut microbiome has been implicated in the modulation of metabolism and linked to nutrition-related chronic diseases such as obesity and diabetes. This lead to hypothesize that lack of a healthy diet may result in impaired immune function during ICIs treatment. Surprisingly, a study shows that obese patients with metastatic melanoma may acquire more benefit from anti-PD therapy than those with normal body mass index (200). Many possible explanations have been mentioned to elucidate this paradox of obesity in cancer, some of which relate to observational biases and the inadequacy of body mass index as an accurate representation of obesity (201).

In summary, gut microbiota may have important implications for the immune response to ICIs and to subsequent development of AKI. Although we are still far from utilization of gut microbiome as ICIs biomarker, the manipulation of commensal bacteria constitution (i.e. by administration of SCFAs) could offer new therapeutic strategies to reduce ICIs-related irAEs and nephrotoxicity (202).

## PD-L1 Overexpression

The PD-L1 overexpression is a strategy of tumor cells to evade immune surveillance. The mechanism of escape is exerted by promoting the T cell exhaustion through PD-1 inhibitory signaling.

Patients with high PD-L1 tumor expression seemed more likely to benefit from anti-PD-1 treatment, although responses were seen even in patients with low or no PD-L1 expression (203, 204).

In several retrospective studies, PD-L1 was the first factor shown to correlate with better outcomes as observed by higher response rate and longer overall survival in melanoma (205) and NSCLC (206). Given these promising results, the PDL-1 expression by immunohistochemistry in tumor biopsy has been considered as one most widely used biomarkers for response to ICIs and has been approved by FDA for patients with NSCLC (112, 207).

However, several concerns still remain for accurate measurement of PD-L1 expression, including different protocols used in each laboratory, the tumor heterogeneity that cannot be represented by the small region of the biopsy sample, the methods for PD-L1 quantization that rely on pathologist evaluations (112). For that reason, additional biomarkers of response to ICIs as the high tumor mutational burden (TMB) are currently assessed and reviewed elsewhere (66, 208).

From a renal perspective, PD-L1 overexpression by renal tubular epithelial cells has been reported in mice model of sepsis-induced AKI (209).

Authors demonstrated that overexpression of PD-L1 is a central mechanism to induce immunosuppression during sepsis, leading to T cell apoptosis and impairment of renal vessel permeability. In addition, they speculated that PD-L1 overexpression could be a potential biomarker to diagnose septic AKI and the treatment with anti-PD-L1 might be a beneficial therapy for septic AKI. In other studies, PD-L1 and PD-L2 have been demonstrated to be involved in AKI and inflammation in a model of bilateral IRI (76). The blocking of PD-L1 and PD-L2 by monoclonal antibodies prior kidney IRI significantly exacerbated the loss of renal function, kidney inflammation and ATIN. A possible explanation could be found in the beneficial PD-L1 /PD-L2 on immunosuppressive Treg able to mediated protection against kidney IRI (77).

In conclusion, PD-L1 signaling appeared to have both a detrimental effect in cancers requiring ICIs or during sepsis, and a beneficial effect on Treg after renal ischemic injury.

However, PD-L1 overexpression on tumor and renal tissue appeared to be a useful biomarker of response to ICIs and the assessment of PD-L1 expression on Treg cells could be important to predict nephrotoxicity during ICIs-induced AKI.

## Author Contributions

RF and ER mainly contributed to the conception, the design, and the writing of the manuscript. GN and FS partly contributed to literature bibliography search. GC and CP critically supported the final draft editing and revised the manuscript. LG and GS revised the final manuscript. RF conceived of all figures. RF took the lead in writing the manuscript, reviewers’ revisions, and figure changes. All authors contributed to the article and approved the submitted version.

## Conflict of Interest

The authors declare that the research was conducted in the absence of any commercial or financial relationships that could be construed as a potential conflict of interest.

## Abbreviations

AIN, acute interstitial nephritis; AKI, acute kidney injury; APC, antigen-presenting cells; ATIN, acute tubulointerstitial nephritis; CKD, chronic kidney disease; CNI, calcineurin inhibitors; CTLA-4, cytotoxic T-lymphocyte antigen 4; ESRD, end-stage renal disease; ICIs, immune checkpoint inhibitors; irAEs, immune related adverse events; IRI, ischemia/reperfusion injury; mTORi, mTOR Inhibitors; NSAID, non steroidal anti-inflammatory drugs; NSCLC, non-small-cell lung cancer; PD-1, programmed cell death protein 1; PD-L1/2, programmed death-ligand 1; PPI, proton pump inhibitors; TCR, T cell receptor.

## References

[1] Yang Y Cancer immunotherapy: harnessing the immune system to battle cancer. J Clin Invest (2015) 125:3335–7. 10.1172/JCI83871 PMC4588312 26325031

[2] Das S Johnson DB Immune-related adverse events and anti-tumor efficacy of immune checkpoint inhibitors. J Immunother Cancer (2019) 7:306. 10.1186/s40425-019-0805-8 31730012 PMC6858629

[3] Jenkins RW Barbie DA Flaherty KT Mechanisms of resistance to immune checkpoint inhibitors. Br J Cancer (2018) 118:9–16. 10.1038/bjc.2017.434 29319049 PMC5765236

[4] Benfaremo D Manfredi L Luchetti MM Gabrielli A Musculoskeletal and Rheumatic Diseases Induced by Immune Checkpoint Inhibitors: A Review of the Literature. Curr Drug Saf (2018) 13:150–64. 10.2174/1574886313666180508122332 PMC6198478 29745339

[5] Perazella MA Shirali AC Immune checkpoint inhibitor nephrotoxicity: what do we know and what should we do? Kidney Int (2020) 97:62–74. 10.1016/j.kint.2019.07.022 31685311

[6] Cortazar FB Kibbelaar ZA Glezerman IG Abudayyeh A Mamlouk O Motwani SS Clinical Features and Outcomes of Immune Checkpoint Inhibitor-Associated AKI: A Multicenter Study. J Am Soc Nephrol (2020) 31:435–46. 10.1681/ASN.2019070676 PMC7003302 31896554

[7] Schneider H Downey J Smith A Zinselmeyer BH Rush C Brewer JM Reversal of the TCR stop signal by CTLA-4. Science (2006) 313:1972–5. 10.1126/science.1131078 16931720

[8] Perez VL Van Parijs L Biuckians A Zheng XX Strom TB Abbas AK Induction of peripheral T cell tolerance in vivo requires CTLA-4 engagement. Immunity (1997) 6:411–7. 10.1016/s1074-7613(00)80284-8 9133420

[9] Linsley PS Golstein P Lymphocyte activation: T-cell regulation by CTLA-4. Curr Biol (1996) 6:398–400. 10.1016/s0960-9822(02)00506-7 8723343

[10] Brunet JF Denizot F Luciani MF Roux-Dosseto M Suzan M Mattei MG A new member of the immunoglobulin superfamily–CTLA-4. Nature (1987) 328:267–70. 10.1038/328267a0 3496540

[11] Wing K Onishi Y Prieto-Martin P Yamaguchi T Miyara M Fehervari Z CTLA-4 control over Foxp3+ regulatory T cell function. Science (2008) 322:271–5. 10.1126/science.1160062 18845758

[12] Chambers CA Sullivan TJ Allison JP Lymphoproliferation in CTLA-4-deficient mice is mediated by costimulation-dependent activation of CD4+ T cells. Immunity (1997) 7:885–95. 10.1016/s1074-7613(00)80406-9 9430233

[13] Chambers CA Kuhns MS Egen JG Allison JP CTLA-4-mediated inhibition in regulation of T cell responses: mechanisms and manipulation in tumor immunotherapy. Annu Rev Immunol (2001) 19:565–94. 10.1146/annurev.immunol.19.1.565 11244047

[14] Leach DR Krummel MF Allison JP Enhancement of antitumor immunity by CTLA-4 blockade. Science (1996) 271:1734–6. 10.1126/science.271.5256.1734 8596936

[15] van Elsas A Hurwitz AA Allison JP Combination immunotherapy of B16 melanoma using anti-cytotoxic T lymphocyte-associated antigen 4 (CTLA-4) and granulocyte/macrophage colony-stimulating factor (GM-CSF)-producing vaccines induces rejection of subcutaneous and metastatic tumors accompanied. J Exp Med (1999) 190:355–66. 10.1084/jem.190.3.355 PMC2195583 10430624

[16] Hurwitz AA Foster BA Kwon ED Truong T Choi EM Greenberg NM Combination immunotherapy of primary prostate cancer in a transgenic mouse model using CTLA-4 blockade. Cancer Res (2000) 60:2444–8. 10811122

[17] Francisco LM Sage PT Sharpe AH The PD-1 pathway in tolerance and autoimmunity. Immunol Rev (2010) 236:219–42. 10.1111/j.1600-065X.2010.00923.x PMC2919275 20636820

[18] Wherry EJ Kurachi M Molecular and cellular insights into T cell exhaustion. Nat Rev Immunol (2015) 15:486–99. 10.1038/nri3862 PMC4889009 26205583

[19] Parsa AT Waldron JS Panner A Crane CA Parney IF Barry JJ Loss of tumor suppressor PTEN function increases B7-H1 expression and immunoresistance in glioma. Nat Med (2007) 13:84–8. 10.1038/nm1517 17159987

[20] Pardoll DM The blockade of immune checkpoints in cancer immunotherapy. Nat Rev Cancer (2012) 12:252–64. 10.1038/nrc3239 PMC4856023 22437870

[21] Ishida Y Agata Y Shibahara K Honjo T Induced expression of PD-1, a novel member of the immunoglobulin gene superfamily, upon programmed cell death. EMBO J (1992) 11:3887–95. 10.1002/j.1460-2075.1992.tb05481.x PMC556898 1396582

[22] Nishimura H Nose M Hiai H Minato N Honjo T Development of lupus-like autoimmune diseases by disruption of the PD-1 gene encoding an ITIM motif-carrying immunoreceptor. Immunity (1999) 11:141–51. 10.1016/s1074-7613(00)80089-8 10485649

[23] Haanen JBAG Robert C Immune Checkpoint Inhibitors. Prog Tumor Res (2015) 42:55–66. 10.1159/000437178 26382943

[24] Yi M Yu S Qin S Liu Q Xu H Zhao W Gut microbiome modulates efficacy of immune checkpoint inhibitors. J Hematol Oncol (2018) 11:47. 10.1186/s13045-018-0592-6 29580257 PMC5870075

[25] Huang R-Y Francois A McGray AR Miliotto A Odunsi K Compensatory upregulation of PD-1, LAG-3, and CTLA-4 limits the efficacy of single-agent checkpoint blockade in metastatic ovarian cancer. Oncoimmunology (2017) 6:e1249561. 10.1080/2162402X.2016.1249561 28197366 PMC5283642

[26] Qin S Xu L Yi M Yu S Wu K Luo S Novel immune checkpoint targets: moving beyond PD-1 and CTLA-4. Mol Cancer (2019) 18:155. 10.1186/s12943-019-1091-2 31690319 PMC6833286

[27] Triebel F Jitsukawa S Baixeras E Roman-Roman S Genevee C Viegas-Pequignot E LAG-3, a novel lymphocyte activation gene closely related to CD4. J Exp Med (1990) 171:1393–405. 10.1084/jem.171.5.1393 PMC2187904 1692078

[28] Kouo T Huang L Pucsek AB Cao M Solt S Armstrong T Galectin-3 Shapes Antitumor Immune Responses by Suppressing CD8+ T Cells via LAG-3 and Inhibiting Expansion of Plasmacytoid Dendritic Cells. Cancer Immunol Res (2015) 3:412–23. 10.1158/2326-6066.CIR-14-0150 PMC4390508 25691328

[29] Mao X Ou MT Karuppagounder SS Kam T-I Yin X Xiong Y Pathological alpha-synuclein transmission initiated by binding lymphocyte-activation gene 3. Science (2016) 353(6307):aah3374. 10.1126/science.aah3374 27708076 PMC5510615

[30] Fourcade J Sun Z Benallaoua M Guillaume P Luescher IF Sander C Upregulation of Tim-3 and PD-1 expression is associated with tumor antigen-specific CD8+ T cell dysfunction in melanoma patients. J Exp Med (2010) 207:2175–86. 10.1084/jem.20100637 PMC2947081 20819923

[31] Koyama S Akbay EA Li YY Herter-Sprie GS Buczkowski KA Richards WG Adaptive resistance to therapeutic PD-1 blockade is associated with upregulation of alternative immune checkpoints. Nat Commun (2016) 7:10501. 10.1038/ncomms10501 26883990 PMC4757784

[32] Fougeray S Brignone C Triebel F A soluble LAG-3 protein as an immunopotentiator for therapeutic vaccines: Preclinical evaluation of IMP321. Vaccine (2006) 24:5426–33. 10.1016/j.vaccine.2006.03.050 16621192

[33] Yu X Huang X Chen X Liu J Wu C Pu Q Characterization of a novel anti-human lymphocyte activation gene 3 (LAG-3) antibody for cancer immunotherapy. MAbs (2019) 11:1139–48. 10.1080/19420862.2019.1629239 PMC6748621 31242068

[34] Postow MA Sidlow R Hellmann MD Immune-Related Adverse Events Associated with Immune Checkpoint Blockade. N Engl J Med (2018) 378:158–68. 10.1056/NEJMra1703481 29320654

[35] Liu RC Consuegra G Chou S Fernandez Penas P Vitiligo-like depigmentation in oncology patients treated with immunotherapies for nonmelanoma metastatic cancers. Clin Exp Dermatol (2019) 44:643–6. 10.1111/ced.13867 30618056

[36] Champiat S Lambotte O Barreau E Belkhir R Berdelou A Carbonnel F Management of immune checkpoint blockade dysimmune toxicities: a collaborative position paper. Ann Oncol (2016) 27:559–74. 10.1093/annonc/mdv623 26715621

[37] De Velasco G Je Y Bosse D Awad MM Ott PA Moreira RB Comprehensive Meta-analysis of Key Immune-Related Adverse Events from CTLA-4 and PD-1/PD-L1 Inhibitors in Cancer Patients. Cancer Immunol Res (2017) 5:312–8. 10.1158/2326-6066.CIR-16-0237 PMC5418853 28246107

[38] Perazella MA Shirali AC Nephrotoxicity of Cancer Immunotherapies: Past, Present and Future. J Am Soc Nephrol (2018) 29:2039–52. 10.1681/ASN.2018050488 PMC6065079 29959196

[39] Cortazar FB Marrone KA Troxell ML Ralto KM Hoenig MP Brahmer JR Clinicopathological features of acute kidney injury associated with immune checkpoint inhibitors. Kidney Int (2016) 90:638–47. 10.1016/j.kint.2016.04.008 PMC4983464 27282937

[40] Manohar S Kompotiatis P Thongprayoon C Cheungpasitporn W Herrmann J Herrmann SM Programmed cell death protein 1 inhibitor treatment is associated with acute kidney injury and hypocalcemia: meta-analysis. Nephrol Dial Transplant (2019) 34:108–17. 10.1093/ndt/gfy105 29762725

[41] Seethapathy H Zhao S Chute DF Zubiri L Oppong Y Strohbehn I The Incidence, Causes, and Risk Factors of Acute Kidney Injury in Patients Receiving Immune Checkpoint Inhibitors. Clin J Am Soc Nephrol (2019) 14:1692–700. 10.2215/CJN.00990119 PMC6895474 31672794

[42] Haslam A Prasad V Estimation of the Percentage of US Patients With Cancer Who Are Eligible for and Respond to Checkpoint Inhibitor Immunotherapy Drugs. JAMA Netw Open (2019) 2:e192535. 10.1001/jamanetworkopen.2019.2535 31050774 PMC6503493

[43] Wanchoo R Karam S Uppal NN Barta VS Deray G Devoe C Adverse Renal Effects of Immune Checkpoint Inhibitors: A Narrative Review. Am J Nephrol (2017) 45:160–9. 10.1159/000455014 28076863

[44] Belliere J Meyer N Mazieres J Ollier S Boulinguez S Delas A Acute interstitial nephritis related to immune checkpoint inhibitors. Br J Cancer (2016) 115:1457–61. 10.1038/bjc.2016.358 PMC5155358 27832664

[45] Mamlouk O Selamet U Machado S Abdelrahim M Glass WF Tchakarov A Nephrotoxicity of immune checkpoint inhibitors beyond tubulointerstitial nephritis: single-center experience. J Immunother Cancer (2019) 7:2. 10.1186/s40425-018-0478-8 30612580 PMC6322290

[46] Marco T Anna P Annalisa T Francesco M Stefania SL Stella D The mechanisms of acute interstitial nephritis in the era of immune checkpoint inhibitors in melanoma. Ther Adv Med Oncol (2019) 11:1758835919875549. 10.1177/1758835919875549 31579108 PMC6759704

[47] Chawla LS Bellomo R Bihorac A Goldstein SL Siew ED Bagshaw SM Acute kidney disease and renal recovery: consensus report of the Acute Disease Quality Initiative (ADQI) 16 Workgroup. Nat Rev Nephrol (2017) 13:241–57. 10.1038/nrneph.2017.2 28239173

[48] Ishitsuka R Miyazaki J Ichioka D Inoue T Kageyama S Sugimoto M Impact of acute kidney injury defined by CTCAE v4.0 during first course of cisplatin-based chemotherapy on treatment outcomes in advanced urothelial cancer patients. Clin Exp Nephrol (2017) 21:732–40. 10.1007/s10157-016-1327-z 27565169

[49] Weber JS Dummer R de Pril V Lebbe C Hodi FS Patterns of onset and resolution of immune-related adverse events of special interest with ipilimumab: detailed safety analysis from a phase 3 trial in patients with advanced melanoma. Cancer (2013) 119:1675–82. 10.1002/cncr.27969 23400564

[50] Weber JS Kahler KC Hauschild A Management of immune-related adverse events and kinetics of response with ipilimumab. J Clin Oncol (2012) 30:2691–7. 10.1200/JCO.2012.41.6750 22614989

[51] Izzedine H Mathian A Champiat S Picard C Mateus C Routier E Renal toxicities associated with pembrolizumab. Clin Kidney J (2019) 12:81–8. 10.1093/ckj/sfy100 PMC6366307 30746132

[52] Ong M Ibrahim AM Bourassa-Blanchette S Canil C Fairhead T Knoll G Antitumor activity of nivolumab on hemodialysis after renal allograft rejection. J Immunother Cancer (2016) 4:64. 10.1186/s40425-016-0171-8 27777773 PMC5067882

[53] Tabei T Natsume I Kobayashi K Successful treatment of metastatic clear cell carcinoma with nivolumab in a patient receiving dialysis treatment. Int J Urol (2017) 24:708–10. 10.1111/iju.13420 28734029

[54] Shirali AC Perazella MA Gettinger S Association of Acute Interstitial Nephritis With Programmed Cell Death 1 Inhibitor Therapy in Lung Cancer Patients. Am J Kidney Dis (2016) 68:287–91. 10.1053/j.ajkd.2016.02.057 27113507

[55] Kidd JM Gizaw AB Ipilimumab-associated minimal-change disease. Kidney Int (2016) 89:720. 10.1016/j.kint.2015.11.028 26880464

[56] Izzedine H Gueutin V Gharbi C Mateus C Robert C Routier E Kidney injuries related to ipilimumab. Invest New Drugs (2014) 32:769–73. 10.1007/s10637-014-0092-7 24687600

[57] Paueksakon P Fogo AB Drug-induced nephropathies. Histopathology (2017) 70:94–108. 10.1111/his.13064 27960238

[58] Kitchlu A Fingrut W Avila-Casado C Chan CT Crump M Hogg D Nephrotic Syndrome With Cancer Immunotherapies: A Report of 2 Cases. Am J Kidney Dis (2017) 70:581–5. 10.1053/j.ajkd.2017.04.026 28648302

[59] Daanen RA Maas RJH Koornstra RHT Steenbergen EJ van Herpen CML Willemsen AECAB Nivolumab-associated Nephrotic Syndrome in a Patient With Renal Cell Carcinoma: A Case Report. J Immunother (2017) 40:345–8. 10.1097/CJI.0000000000000189 28961608

[60] Jung K Zeng X Bilusic M Nivolumab-associated acute glomerulonephritis: a case report and literature review. BMC Nephrol (2016) 17:188. 10.1186/s12882-016-0408-2 27876011 PMC5120473

[61] Kim SH Park SJ Han KH Kronbichler A Saleem MA Oh J Shin J Il. Pathogenesis of minimal change nephrotic syndrome: an immunological concept. Korean J Pediatr (2016) 59:205–11. 10.3345/kjp.2016.59.5.205 PMC4897155 27279884

[62] Blank M-L Parkin L Paul C Herbison P A nationwide nested case-control study indicates an increased risk of acute interstitial nephritis with proton pump inhibitor use. Kidney Int (2014) 86:837–44. 10.1038/ki.2014.74 PMC4184187 24646856

[63] Intlekofer AM Thompson CB At the bench: preclinical rationale for CTLA-4 and PD-1 blockade as cancer immunotherapy. J Leukoc Biol (2013) 94:25–39. 10.1189/jlb.1212621 23625198 PMC3685017

[64] Wolchok JD Kluger H Callahan MK Postow MA Rizvi NA Lesokhin AM Nivolumab plus ipilimumab in advanced melanoma. N Engl J Med (2013) 369:122–33. 10.1056/NEJMoa1302369 PMC5698004 23724867

[65] Motzer RJ Tannir NM McDermott DF Aren Frontera O Melichar B Choueiri TK Nivolumab plus Ipilimumab versus Sunitinib in Advanced Renal-Cell Carcinoma. N Engl J Med (2018) 378:1277–90. 10.1056/NEJMoa1712126 PMC5972549 29562145

[66] Hellmann MD Paz-Ares L Bernabe Caro R Zurawski B Kim S-W Carcereny Costa E Nivolumab plus Ipilimumab in Advanced Non-Small-Cell Lung Cancer. N Engl J Med (2019) 381:2020–31. 10.1056/NEJMoa1910231 31562796

[67] Abdel-Wahab N Shah M Suarez-Almazor ME Adverse Events Associated with Immune Checkpoint Blockade in Patients with Cancer: A Systematic Review of Case Reports. PLoS One (2016) 11:e0160221. 10.1371/journal.pone.0160221 27472273 PMC4966895

[68] Fadel F El Karoui K Knebelmann B Anti-CTLA4 antibody-induced lupus nephritis. N Engl J Med (2009) 361:211–2. 10.1056/NEJMc0904283 19587352

[69] Johnson DB Balko JM Compton ML Chalkias S Gorham J Xu Y Fulminant Myocarditis with Combination Immune Checkpoint Blockade. N Engl J Med (2016) 375:1749–55. 10.1056/NEJMoa1609214 PMC5247797 27806233

[70] Zehn D Bevan MJ T cells with low avidity for a tissue-restricted antigen routinely evade central and peripheral tolerance and cause autoimmunity. Immunity (2006) 25:261–70. 10.1016/j.immuni.2006.06.009 PMC2774714 16879996

[71] Richards DM Kyewski B Feuerer M Re-examining the Nature and Function of Self-Reactive T cells. Trends Immunol (2016) 37:114–25. 10.1016/j.it.2015.12.005 PMC7611850 26795134

[72] Ding H Wu X Gao W PD-L1 is expressed by human renal tubular epithelial cells and suppresses T cell cytokine synthesis. Clin Immunol (2005) 115:184–91. 10.1016/j.clim.2005.01.005 15885642

[73] Schoop R Wahl P Le Hir M Heemann U Wang M Wuthrich RP Suppressed T-cell activation by IFN-gamma-induced expression of PD-L1 on renal tubular epithelial cells. Nephrol Dial Transplant (2004) 19:2713–20. 10.1093/ndt/gfh423 15353579

[74] Murakami N Motwani S Riella LV Renal complications of immune checkpoint blockade. Curr Probl Cancer (2017) 41:100–10. 10.1016/j.currproblcancer.2016.12.004 PMC5440194 28189263

[75] Murakami N Borges TJ Yamashita M Riella LV Severe acute interstitial nephritis after combination immune-checkpoint inhibitor therapy for metastatic melanoma. Clin Kidney J (2016) 9:411–7. 10.1093/ckj/sfw024 PMC4886917 27274826

[76] Jaworska K Ratajczak J Huang L Whalen K Yang M Stevens BK Both PD-1 ligands protect the kidney from ischemia reperfusion injury. J Immunol (2015) 194:325–33. 10.4049/jimmunol.1400497 PMC4272874 25404361

[77] Kinsey GR Huang L Jaworska K Khutsishvili K Becker DA Ye H Autocrine adenosine signaling promotes regulatory T cell-mediated renal protection. J Am Soc Nephrol (2012) 23:1528–37. 10.1681/ASN.2012010070 PMC3431416 22835488

[78] Koda R Watanabe H Tsuchida M Iino N Suzuki K Hasegawa G Immune checkpoint inhibitor (nivolumab)-associated kidney injury and the importance of recognizing concomitant medications known to cause acute tubulointerstitial nephritis: a case report. BMC Nephrol (2018) 19:48. 10.1186/s12882-018-0848-y 29486725 PMC5830324

[79] Gigante M Blasi A Loverre A Mancini V Battaglia M Selvaggi FP Dysfunctional DC subsets in RCC patients: ex vivo correction to yield an effective anti-cancer vaccine. Mol Immunol (2009) 46:893–901. 10.1016/j.molimm.2008.09.015 19041139 PMC3427923

[80] Dimitriou F Matter AV Mangana J Urosevic-Maiwald M Micaletto S Braun RP Cytokine Release Syndrome During Sequential Treatment With Immune Checkpoint Inhibitors and Kinase Inhibitors for Metastatic Melanoma. J Immunother (2019) 42:29–32. 10.1097/CJI.0000000000000236 29939877

[81] Breda A Lucarelli G Rodriguez-Faba O Guirado L Facundo C Bettocchi C Erratum to: Clinical and pathological outcomes of renal cell carcinoma (RCC) in native kidneys of patients with end-stage renal disease: a long-term comparative retrospective study with RCC diagnosed in the general population. World J Urol (2015) 33:9. 10.1007/s00345-014-1268-7 24577798

[82] Engels EA Pfeiffer RM Fraumeni JFJ Kasiske BL Israni AK Snyder JJ Spectrum of cancer risk among US solid organ transplant recipients. JAMA (2011) 306:1891–901. 10.1001/jama.2011.1592 PMC3310893 22045767

[83] Triplette M Crothers K Mahale P Yanik EL Valapour M Lynch CF Risk of lung cancer in lung transplant recipients in the United States. Am J Transplant (2019) 19:1478–90. 10.1111/ajt.15181 PMC6872188 30565414

[84] Stallone G Schena A Infante B Di Paolo S Loverre A Maggio G Sirolimus for Kaposi’s sarcoma in renal-transplant recipients. N Engl J Med (2005) 352:1317–23. 10.1056/NEJMoa042831 15800227

[85] Penn I Evaluation of transplant candidates with pre-existing malignancies. Ann Transplant (1997) 2:14–7. 9869873

[86] Watschinger B Budde K Crespo M Heemann U Hilbrands L Maggiore U Pre-existing malignancies in renal transplant candidates-time to reconsider waiting times. Nephrol Dial Transplant Off Publ Eur Dial Transpl Assoc - Eur Ren Assoc (2019) 34:1292–300. 10.1093/ndt/gfz026 30830155

[87] Venkatachalam K Malone AF Heady B Delos Santos R Alhamad T Poor Outcomes with the Use of Checkpoint Inhibitors in Kidney Transplant Recipients. Transplantation (2019) 104(5):1041–47. 10.1097/TP.0000000000002914 31415036

[88] Murakami N Riella LV Co-inhibitory pathways and their importance in immune regulation. Transplantation (2014) 98:3–14. 10.1097/TP.0000000000000169 24978034

[89] Lai H-C Lin J-F Hwang TIS Liu Y-F Yang A-H Wu C-K Programmed Cell Death 1 (PD-1) Inhibitors in Renal Transplant Patients with Advanced Cancer: A Double-Edged Sword? Int J Mol Sci (2019) 20(9):2194. 10.3390/ijms20092194 PMC6540260 31058839

[90] Escandon J Peacock S Trabolsi A Thomas DB Layka A Lutzky J Interstitial nephritis in melanoma patients secondary to PD-1 checkpoint inhibitor. J Immunother Cancer (2017) 5:3. 10.1186/s40425-016-0205-2 28105370 PMC5240303

[91] Alhamad T Venkatachalam K Linette GP Brennan DC Checkpoint Inhibitors in Kidney Transplant Recipients and the Potential Risk of Rejection. Am J Transplant (2016) 16:1332–3. 10.1111/ajt.13711 26752406

[92] Deltombe C Garandeau C Renaudin K Hourmant M Severe Allograft Rejection and Autoimmune Hemolytic Anemia After Anti-PD1 Therapy in a Kidney Transplanted Patient. Transplantation (2017) 101:e291. 10.1097/TP.0000000000001861 29633980

[93] Boils CL Aljadir DN Cantafio AW Use of the PD-1 Pathway Inhibitor Nivolumab in a Renal Transplant Patient With Malignancy. Am J Transplant (2016) 16:2496–7. 10.1111/ajt.13786 26988410

[94] Tamain M Garrouste C Aguilera D Tiple A Salhi S Kosmadakis G Mixed acute kidney allograft rejection after an antiprogrammed cell death protein 1 antibody treatment for lung epidermoid carcinoma. Transpl Int (2016) 29:1247–8. 10.1111/tri.12834 27529458

[95] Stallone G Infante B Pontrelli P Gigante M Montemurno E Loverre A Sirolimus and proteinuria in renal transplant patients: evidence for a dose-dependent effect on slit diaphragm-associated proteins. Transplantation (2011) 91:997–1004. 10.1097/TP.0b013e318211d342 21364499

[96] Stallone G Infante B Grandaliano G Management and prevention of post-transplant malignancies in kidney transplant recipients. Clin Kidney J (2015) 8:637–44. 10.1093/ckj/sfv054 PMC4581374 26413294

[97] Lim WH Eris J Kanellis J Pussell B Wiid Z Witcombe D A systematic review of conversion from calcineurin inhibitor to mammalian target of rapamycin inhibitors for maintenance immunosuppression in kidney transplant recipients. Am J Transplant (2014) 14:2106–19. 10.1111/ajt.12795 25088685

[98] Dantal J Morelon E Rostaing L Goffin E Brocard A Tromme I Sirolimus for Secondary Prevention of Skin Cancer in Kidney Transplant Recipients: 5-Year Results. J Clin Oncol (2018) 36:2612–20. 10.1200/JCO.2017.76.6691 30016177

[99] Knoll GA Kokolo MB Mallick R Beck A Buenaventura CD Ducharme R Effect of sirolimus on malignancy and survival after kidney transplantation: systematic review and meta-analysis of individual patient data. BMJ (2014) 349:g6679. 10.1136/bmj.g6679 25422259 PMC4241732

[100] Kauffman HM Cherikh WS Cheng Y Hanto DW Kahan BD Maintenance immunosuppression with target-of-rapamycin inhibitors is associated with a reduced incidence of de novo malignancies. Transplantation (2005) 80:883–9. 10.1097/01.tp.0000184006.43152.8d 16249734

[101] Euvrard S Morelon E Rostaing L Goffin E Brocard A Tromme I Sirolimus and secondary skin-cancer prevention in kidney transplantation. N Engl J Med (2012) 367:329–39. 10.1056/NEJMoa1204166 22830463

[102] Tian T Li X Zhang J mTOR Signaling in Cancer and mTOR Inhibitors in Solid Tumor Targeting Therapy. Int J Mol Sci (2019) 20(3):755. 10.3390/ijms20030755 PMC6387042 30754640

[103] Hua H Kong Q Zhang H Wang J Luo T Jiang Y Targeting mTOR for cancer therapy. J Hematol Oncol (2019) 12:71. 10.1186/s13045-019-0754-1 31277692 PMC6612215

[104] Procaccini C De Rosa V Galgani M Abanni L Calì G Porcellini A An oscillatory switch in mTOR kinase activity sets regulatory T cell responsiveness. Immunity (2010) 33:929–41. 10.1016/j.immuni.2010.11.024 PMC3133602 21145759

[105] Sabbatini M Ruggiero G Palatucci AT Rubino V Federico S Giovazzino A Oscillatory mTOR inhibition and Treg increase in kidney transplantation. Clin Exp Immunol (2015) 182:230–40. 10.1111/cei.12669 PMC4608513 26077103

[106] Rousseau B Guillemin A Duvoux C Neuzillet C Tlemsani C Compagnon P Optimal oncologic management and mTOR inhibitor introduction are safe and improve survival in kidney and liver allograft recipients with de novo carcinoma. Int J Cancer (2019) 144:886–96. 10.1002/ijc.31769 30155929

[107] Vanasek TL Khoruts A Zell T Mueller DL Antagonistic roles for CTLA-4 and the mammalian target of rapamycin in the regulation of clonal anergy: enhanced cell cycle progression promotes recall antigen responsiveness. J Immunol (2001) 167:5636–44. 10.4049/jimmunol.167.10.5636 11698435

[108] Longhi MS Ma Y Bogdanos DP Cheeseman P Mieli-Vergani G Vergani D Impairment of CD4(+)CD25(+) regulatory T-cells in autoimmune liver disease. J Hepatol (2004) 41:31–7. 10.1016/j.jhep.2004.03.008 15246204

[109] Stallone G Infante B Di Lorenzo A Rascio F Zaza G Grandaliano G mTOR inhibitors effects on regulatory T cells and on dendritic cells. J Transl Med (2016) 14:152. 10.1186/s12967-016-0916-7 27245075 PMC4886438

[110] Barnett R Barta VS Jhaveri KD Preserved Renal-Allograft Function and the PD-1 Pathway Inhibitor Nivolumab. N Engl J Med (2017) 376:191–2. 10.1056/NEJMc1614298 28076715

[111] Esfahani K Al-Aubodah T-A Thebault P Lapointe R Hudson M Johnson NA Targeting the mTOR pathway uncouples the efficacy and toxicity of PD-1 blockade in renal transplantation. Nat Commun (2019) 10:4712. 10.1038/s41467-019-12628-1 31624262 PMC6797722

[112] Havel JJ Chowell D Chan TA The evolving landscape of biomarkers for checkpoint inhibitor immunotherapy. Nat Rev Cancer (2019) 19:133–50. 10.1038/s41568-019-0116-x PMC6705396 30755690

[113] Nakamura Y Biomarkers for Immune Checkpoint Inhibitor-Mediated Tumor Response and Adverse Events. Front Med (2019) 6:119. 10.3389/fmed.2019.00119 PMC6549005 31192215

[114] Conforti F Pala L Bagnardi V De Pas T Martinetti M Viale G Cancer immunotherapy efficacy and patients’ sex: a systematic review and meta-analysis. Lancet Oncol (2018) 19:737–46. 10.1016/S1470-2045(18)30261-4 29778737

[115] Valpione S Pasquali S Campana LG Piccin L Mocellin S Pigozzo J Sex and interleukin-6 are prognostic factors for autoimmune toxicity following treatment with anti-CTLA4 blockade. J Transl Med (2018) 16:94. 10.1186/s12967-018-1467-x 29642948 PMC5896157

[116] Snyder A Makarov V Merghoub T Yuan J Zaretsky JM Desrichard A Genetic basis for clinical response to CTLA-4 blockade in melanoma. N Engl J Med (2014) 371:2189–99. 10.1056/NEJMoa1406498 PMC4315319 25409260

[117] Samstein RM Lee C-H Shoushtari AN Hellmann MD Shen R Janjigian YY Tumor mutational load predicts survival after immunotherapy across multiple cancer types. Nat Genet (2019) 51:202–6. 10.1038/s41588-018-0312-8 PMC6365097 30643254

[118] Routy B Le Chatelier E Derosa L Duong CPM Alou MT Daillere R Gut microbiome influences efficacy of PD-1-based immunotherapy against epithelial tumors. Science (2018) 359:91–7. 10.1126/science.aan3706 29097494

[119] Hou Q Xu H Rational Discovery of Response Biomarkers: Candidate Prognostic Factors and Biomarkers for Checkpoint Inhibitor-Based Immunotherapy. Adv Exp Med Biol (2020) 1248:143–66. 10.1007/978-981-15-3266-5_7 32185710

[120] Carbone DP Reck M Paz-Ares L Creelan B Horn L Steins M First-Line Nivolumab in Stage IV or Recurrent Non-Small-Cell Lung Cancer. N Engl J Med (2017) 376:2415–26. 10.1056/NEJMoa1613493 PMC6487310 28636851

[121] Garon EB Cancer Immunotherapy Trials Not Immune from Imprecise Selection of Patients. N Engl J Med (2017) 376:2483–5. 10.1056/NEJMe1705692 28636845

[122] Lucarelli G Loizzo D Franzin R Battaglia S Ferro M Cantiello F Metabolomic insights into pathophysiological mechanisms and biomarker discovery in clear cell renal cell carcinoma. Expert Rev Mol Diagn (2019) 19:397–407. 10.1080/14737159.2019.1607729 30983433

[123] Martens A Wistuba-Hamprecht K Geukes Foppen M Yuan J Postow MA Wong P Baseline Peripheral Blood Biomarkers Associated with Clinical Outcome of Advanced Melanoma Patients Treated with Ipilimumab. Clin Cancer Res (2016) 22:2908–18. 10.1158/1078-0432.CCR-15-2412 PMC5770142 26787752

[124] Nakamura Y Tanaka R Maruyama H Ishitsuka Y Okiyama N Watanabe R Correlation between blood cell count and outcome of melanoma patients treated with anti-PD-1 antibodies. Jpn J Clin Oncol (2019) 49:431–7. 10.1093/jjco/hyy201 30753621

[125] Weide B Martens A Hassel JC Berking C Postow MA Bisschop K Baseline Biomarkers for Outcome of Melanoma Patients Treated with Pembrolizumab. Clin Cancer Res (2016) 22:5487–96. 10.1158/1078-0432.CCR-16-0127 PMC5572569 27185375

[126] Diehl A Yarchoan M Hopkins A Jaffee E Grossman SA Relationships between lymphocyte counts and treatment-related toxicities and clinical responses in patients with solid tumors treated with PD-1 checkpoint inhibitors. Oncotarget (2017) 8:114268–80. 10.18632/oncotarget.23217 PMC5768402 29371985

[127] Abraham C Cho J Interleukin-23/Th17 pathways and inflammatory bowel disease. Inflammation Bowel Dis (2009) 15:1090–100. 10.1002/ibd.20894 19253307

[128] Tarhini AA Zahoor H Lin Y Malhotra U Sander C Butterfield LH Baseline circulating IL-17 predicts toxicity while TGF-beta1 and IL-10 are prognostic of relapse in ipilimumab neoadjuvant therapy of melanoma. J Immunother Cancer (2015) 3:39. 10.1186/s40425-015-0081-1 26380086 PMC4570556

[129] von Euw E Chodon T Attar N Jalil J Koya RC Comin-Anduix B CTLA4 blockade increases Th17 cells in patients with metastatic melanoma. J Transl Med (2009) 7:35. 10.1186/1479-5876-7-35 19457253 PMC2697137

[130] Loverre A Divella C Castellano G Tataranni T Zaza G Rossini M T helper 1, 2 and 17 cell subsets in renal transplant patients with delayed graft function. Transpl Int Off J Eur Soc Organ Transplant (2011) 24:233–42. 10.1111/j.1432-2277.2010.01157.x 21281362

[131] Maravitsa P Adamopoulou M Pistiki A Netea MG Louis K Giamarellos-Bourboulis EJ Systemic over-release of interleukin-17 in acute kidney injury after septic shock: Clinical and experimental evidence. Immunol Lett (2016) 178:68–76. 10.1016/j.imlet.2016.08.002 27515003

[132] Mehrotra P Collett JA Gunst SJ Basile DP Th17 cells contribute to pulmonary fibrosis and inflammation during chronic kidney disease progression after acute ischemia. Am J Physiol Regul Integr Comp Physiol (2018) 314:R265–73. 10.1152/ajpregu.00147.2017 PMC5867669 29118018

[133] Jeong H Hwang I Kang SH Shin HC Kwon SY Tumor-Associated Macrophages as Potential Prognostic Biomarkers of Invasive Breast Cancer. J Breast Cancer (2019) 22:38–51. 10.4048/jbc.2019.22.e5 30941232 PMC6438840

[134] Fujimura T Kakizaki A Furudate S Aiba S A possible interaction between periostin and CD163(+) skin-resident macrophages in pemphigus vulgaris and bullous pemphigoid. Exp Dermatol (2017) 26:1193–8. 10.1111/exd.13157 27501402

[135] Fujimura T Sato Y Tanita K Kambayashi Y Otsuka A Fujisawa Y Serum levels of soluble CD163 and CXCL5 may be predictive markers for immune-related adverse events in patients with advanced melanoma treated with nivolumab: a pilot study. Oncotarget (2018) 9:15542–51. 10.18632/oncotarget.24509 PMC5884646 29643991

[136] Chen T Cao Q Wang Y Harris DCH M2 macrophages in kidney disease: biology, therapies, and perspectives. Kidney Int (2019) 95:760–73. 10.1016/j.kint.2018.10.041 30827512

[137] Su L Feng L Liu C Jiang Z Li M Xiao K Diagnostic value of urine sCD163 levels for sepsis and relevant acute kidney injury: a prospective study. BMC Nephrol (2012) 13:123. 10.1186/1471-2369-13-123 23013330 PMC3506529

[138] Sun P-P Zhou X-J Su J-Q Wang C Yu X-J Su T Urine macrophages reflect kidney macrophage content during acute tubular interstitial and glomerular injury. Clin Immunol (2019) 205:65–74. 10.1016/j.clim.2019.06.005 31212026

[139] Kim M-G Lim K Lee YJ Yang J Oh SW Cho WY M2 macrophages predict worse long-term outcomes in human acute tubular necrosis. Sci Rep (2020) 10:2122. 10.1038/s41598-020-58725-w 32034190 PMC7005727

[140] Jeurling S Cappelli LC Treatment of immune checkpoint inhibitor-induced inflammatory arthritis. Curr Opin Rheumatol (2020) 32:315–20. 10.1097/BOR.0000000000000701 PMC7212600 32168068

[141] Tanaka R Okiyama N Okune M Ishitsuka Y Watanabe R Furuta J Serum level of interleukin-6 is increased in nivolumab-associated psoriasiform dermatitis and tumor necrosis factor-alpha is a biomarker of nivolumab recativity. J Dermatol Sci (2017) 86:71–3. 10.1016/j.jdermsci.2016.12.019 28069323

[142] Husain-Syed F Slutsky AS Ronco C Lung-Kidney Cross-Talk in the Critically Ill Patient. Am J Respir Crit Care Med (2016) 194:402–14. 10.1164/rccm.201602-0420CP 27337068

[143] Jordan SC Choi J Kim I Wu G Toyoda M Shin B Interleukin-6, A Cytokine Critical to Mediation of Inflammation, Autoimmunity and Allograft Rejection: Therapeutic Implications of IL-6 Receptor Blockade. Transplantation (2017) 101:32–44. 10.1097/TP.0000000000001452 27547870

[144] Zhang WR Garg AX Coca SG Devereaux PJ Eikelboom J Kavsak P Plasma IL-6 and IL-10 Concentrations Predict AKI and Long-Term Mortality in Adults after Cardiac Surgery. J Am Soc Nephrol (2015) 26:3123–32. 10.1681/ASN.2014080764 PMC4657830 25855775

[145] Liu KD Altmann C Smits G Krawczeski CD Edelstein CL Devarajan P Serum interleukin-6 and interleukin-8 are early biomarkers of acute kidney injury and predict prolonged mechanical ventilation in children undergoing cardiac surgery: a case-control study. Crit Care (2009) 13:R104. 10.1186/cc7940 19570208 PMC2750143

[146] Wu C Chen X Cai Y Xia J Zhou X Xu S Risk Factors Associated With Acute Respiratory Distress Syndrome and Death in Patients With Coronavirus Disease 2019 Pneumonia in Wuhan, China. JAMA Intern Med (2020) 180(7):934–43. 10.1001/jamainternmed.2020.0994 PMC7070509 32167524

[147] Gershenwald JE Scolyer RA Hess KR Sondak VK Long GV Ross MI Melanoma staging: Evidence-based changes in the American Joint Committee on Cancer eighth edition cancer staging manual. CA Cancer J Clin (2017) 67:472–92. 10.3322/caac.21409 PMC5978683 29028110

[148] Agarwala SS Keilholz U Gilles E Bedikian AY Wu J Kay R LDH correlation with survival in advanced melanoma from two large, randomised trials (Oblimersen GM301 and EORTC 18951). Eur J Cancer (2009) 45:1807–14. 10.1016/j.ejca.2009.04.016 19419855

[149] Shen J Chen Z Zhuang Q Fan M Ding T Lu H Prognostic Value of Serum Lactate Dehydrogenase in Renal Cell Carcinoma: A Systematic Review and Meta-Analysis. PLoS One (2016) 11:e0166482. 10.1371/journal.pone.0166482 27861542 PMC5115746

[150] Delyon J Mateus C Lefeuvre D Lanoy E Zitvogel L Chaput N Experience in daily practice with ipilimumab for the treatment of patients with metastatic melanoma: an early increase in lymphocyte and eosinophil counts is associated with improved survival. Ann Oncol Off J Eur Soc Med Oncol (2013) 24:1697–703. 10.1093/annonc/mdt027 23439861

[151] Kelderman S Heemskerk B van Tinteren H van den Brom RRH Hospers GAP van den Eertwegh AJM Lactate dehydrogenase as a selection criterion for ipilimumab treatment in metastatic melanoma. Cancer Immunol Immunother (2014) 63:449–58. 10.1007/s00262-014-1528-9 PMC11029318 24609989

[152] Diem S Kasenda B Spain L Martin-Liberal J Marconcini R Gore M Serum lactate dehydrogenase as an early marker for outcome in patients treated with anti-PD-1 therapy in metastatic melanoma. Br J Cancer (2016) 114:256–61. 10.1038/bjc.2015.467 PMC4742588 26794281

[153] Taniguchi Y Tamiya A Isa S-I Nakahama K Okishio K Shiroyama T Predictive Factors for Poor Progression-free Survival in Patients with Non-small Cell Lung Cancer Treated with Nivolumab. Anticancer Res (2017) 37:5857–62. 10.21873/anticanres.12030 28982912

[154] Won AJ Kim S Kim YG Kim K-B Choi WS Kacew S Discovery of urinary metabolomic biomarkers for early detection of acute kidney injury. Mol Biosyst (2016) 12:133–44. 10.1039/c5mb00492f 26566257

[155] Alzahri MS Mousa SA Almomen AM Hasanato RM Polimeni JM Racz MJ Lactate dehydrogenase as a biomarker for early renal damage in patients with sickle cell disease. Saudi J Kidney Dis Transpl (2015) 26:1161–8. 10.4103/1319-2442.168596 26586054

[156] Westhuyzen J Endre ZH Reece G Reith DM Saltissi D Morgan TJ Measurement of tubular enzymuria facilitates early detection of acute renal impairment in the intensive care unit. Nephrol Dial Transplant (2003) 18:543–51. 10.1093/ndt/18.3.543 12584277

[157] Herget-Rosenthal S Poppen D Husing J Marggraf G Pietruck F Jakob H-G Prognostic value of tubular proteinuria and enzymuria in nonoliguric acute tubular necrosis. Clin Chem (2004) 50:552–8. 10.1373/clinchem.2003.027763 14709451

[158] Duggal NA Reversing the immune ageing clock: lifestyle modifications and pharmacological interventions. Biogerontology (2018) 19:481–96. 10.1007/s10522-018-9771-7 PMC6223743 30269199

[159] Goronzy JJ Weyand CM Understanding immunosenescence to improve responses to vaccines. Nat Immunol (2013) 14:428–36. 10.1038/ni.2588 PMC4183346 23598398

[160] Franzin R Stasi A Fiorentino M Stallone G Cantaluppi V Gesualdo L Inflammaging and Complement System: A Link Between Acute Kidney Injury and Chronic Graft Damage. Front Immunol (2020) 11:734. 10.3389/fimmu.2020.00734 32457738 PMC7221190

[161] Duggal NA Niemiro G Harridge SDR Simpson RJ Lord JM Can physical activity ameliorate immunosenescence and thereby reduce age-related multi-morbidity? Nat Rev Immunol (2019) 19:563–72. 10.1038/s41577-019-0177-9 31175337

[162] Losappio V Franzin R Infante B Godeas G Gesualdo L Fersini A Molecular Mechanisms of Premature Aging in Hemodialysis: The Complex Interplay Between Innate and Adaptive Immune Dysfunction. Int J Mol Sci (2020) 21(10):3422. 10.3390/ijms21103422 PMC7279398 32408613

[163] Li P Yang X Feng Y Wu L Ma W Ding G The impact of immunosenescence on the efficacy of immune checkpoint inhibitors in melanoma patients: a meta-analysis. Onco Targets Ther (2018) 11:7521–7. 10.2147/OTT.S165368 PMC6208870 30464500

[164] Moreira A Gross S Kirchberger MC Erdmann M Schuler G Heinzerling L Senescence markers: Predictive for response to checkpoint inhibitors. Int J Cancer (2019) 144:1147–50. 10.1002/ijc.31763 30151962

[165] Pawelec G Hallmarks of human “immunosenescence”: adaptation or dysregulation? Immun Ageing (2012) 9:15. 10.1186/1742-4933-9-15 22830639 PMC3416738

[166] Zaretsky JM Garcia-Diaz A Shin DS Escuin-Ordinas H Hugo W Hu-Lieskovan S Mutations Associated with Acquired Resistance to PD-1 Blockade in Melanoma. N Engl J Med (2016) 375:819–29. 10.1056/NEJMoa1604958 PMC5007206 27433843

[167] Braumuller H Wieder T Brenner E Assmann S Hahn M Alkhaled M T-helper-1-cell cytokines drive cancer into senescence. Nature (2013) 494:361–5. 10.1038/nature11824 23376950

[168] Gigante M Mandic M Wesa AK Cavalcanti E Dambrosio M Mancini V Interferon-alpha (IFN-alpha)-conditioned DC preferentially stimulate type-1 and limit Treg-type in vitro T-cell responses from RCC patients. J Immunother (2008) 31:254–62. 10.1097/CJI.0b013e318167b023 18317362

[169] Hodgson LE Venn RM Short S Roderick PJ Hargreaves D Selby N Improving clinical prediction rules in acute kidney injury with the use of biomarkers of cell cycle arrest: a pilot study. Biomarkers Biochem Indic Expo Response Susceptibility to Chem (2019) 24:23–8. 10.1080/1354750X.2018.1493617 29943653

[170] Ortega LM Heung M The use of cell cycle arrest biomarkers in the early detection of acute kidney injury. Is this the new renal troponin? Nefrologia (2018) 38:361–7. 10.1016/j.nefro.2017.11.013 29627229

[171] Peng Z-Y Zhou F Kellum JA Cross-species validation of cell cycle arrest markers for acute kidney injury in the rat during sepsis. Intensive Care Med Exp (2016) 4:12. 10.1186/s40635-016-0086-1 27245788 PMC4887455

[172] Srisawat N Kellum JA The Role of Biomarkers in Acute Kidney Injury. Crit Care Clin (2020) 36:125–40. 10.1016/j.ccc.2019.08.010 31733675

[173] Joannidis M Forni LG Haase M Koyner J Shi J Kashani K Use of Cell Cycle Arrest Biomarkers in Conjunction With Classical Markers of Acute Kidney Injury. Crit Care Med (2019) 47:e820–6. 10.1097/CCM.0000000000003907 PMC6750148 31343478

[174] Kashani K Al-Khafaji A Ardiles T Artigas A Bagshaw SM Bell M Discovery and validation of cell cycle arrest biomarkers in human acute kidney injury. Crit Care (2013) 17:R25. 10.1186/cc12503 23388612 PMC4057242

[175] Gigante M Lucarelli G Divella C Netti GS Pontrelli P Cafiero C Soluble Serum αKlotho Is a Potential Predictive Marker of Disease Progression in Clear Cell Renal Cell Carcinoma. Med (Baltimore) (2015) 94:e1917. 10.1097/MD.0000000000001917 PMC4912252 26559258

[176] Melk A Schmidt BMW Vongwiwatana A Rayner DC Halloran PF Increased expression of senescence-associated cell cycle inhibitor p16INK4a in deteriorating renal transplants and diseased native kidney. Am J Transplant (2005) 5:1375–82. 10.1111/j.1600-6143.2005.00846.x 15888044

[177] Castellano G Franzin R Sallustio F Stasi A Banelli B Romani M Complement component C5a induces aberrant epigenetic modifications in renal tubular epithelial cells accelerating senescence by Wnt4/betacatenin signaling after ischemia/reperfusion injury. Aging (Albany NY) (2019) 11:4382–406. 10.18632/aging.102059 PMC6660044 31284268

[178] Castellano G Intini A Stasi A Divella C Gigante M Pontrelli P Complement Modulation of Anti-Aging Factor Klotho in Ischemia/Reperfusion Injury and Delayed Graft Function. Am J Transplant (2016) 16:325–33. 10.1111/ajt.13415 26280899

[179] Johnson AC Zager RA Plasma and urinary p21: potential biomarkers of AKI and renal aging. Am J Physiol Renal Physiol (2018) 315:F1329–35. 10.1152/ajprenal.00328.2018 PMC6293288 30066587

[180] Fiorentino M Grandaliano G Gesualdo L Castellano G Acute Kidney Injury to Chronic Kidney Disease Transition. Contrib Nephrol (2018) 193:45–54. 10.1159/000484962 29393158

[181] Kho ZY Lal SK The Human Gut Microbiome - A Potential Controller of Wellness and Disease. Front Microbiol (2018) 9:1835. 10.3389/fmicb.2018.01835 30154767 PMC6102370

[182] Bhatt AP Redinbo MR Bultman SJ The role of the microbiome in cancer development and therapy. CA Cancer J Clin (2017) 67:326–44. 10.3322/caac.21398 PMC5530583 28481406

[183] Chaput N Lepage P Coutzac C Soularue E Le Roux K Monot C Baseline gut microbiota predicts clinical response and colitis in metastatic melanoma patients treated with ipilimumab. Ann Oncol (2017) 28:1368–79. 10.1093/annonc/mdx108 28368458

[184] Matson V Fessler J Bao R Chongsuwat T Zha Y Alegre M-L The commensal microbiome is associated with anti-PD-1 efficacy in metastatic melanoma patients. Science (2018) 359:104–8. 10.1126/science.aao3290 PMC6707353 29302014

[185] Gopalakrishnan V Spencer CN Nezi L Reuben A Andrews MC Karpinets TV Gut microbiome modulates response to anti-PD-1 immunotherapy in melanoma patients. Science (2018) 359:97–103. 10.1126/science.aan4236 29097493 PMC5827966

[186] Derosa L Routy B Fidelle M Iebba V Alla L Pasolli E Gut Bacteria Composition Drives Primary Resistance to Cancer Immunotherapy in Renal Cell Carcinoma Patients. Eur Urol (2020) 78(2):195–206. 10.1016/j.eururo.2020.04.044 32376136

[187] Dubin K Callahan MK Ren B Khanin R Viale A Ling L Intestinal microbiome analyses identify melanoma patients at risk for checkpoint-blockade-induced colitis. Nat Commun (2016) 7:10391. 10.1038/ncomms10391 26837003 PMC4740747

[188] Yacoub R Wyatt CM Manipulating the gut microbiome to decrease uremic toxins. Kidney Int (2017) 91:521–3. 10.1016/j.kint.2017.01.003 28202164

[189] Mishima E Fukuda S Mukawa C Yuri A Kanemitsu Y Matsumoto Y Evaluation of the impact of gut microbiota on uremic solute accumulation by a CE-TOFMS-based metabolomics approach. Kidney Int (2017) 92:634–45. 10.1016/j.kint.2017.02.011 28396122

[190] Niwa T Role of indoxyl sulfate in the progression of chronic kidney disease and cardiovascular disease: experimental and clinical effects of oral sorbent AST-120. Ther Apher Dial (2011) 15:120–4. 10.1111/j.1744-9987.2010.00882.x 21426500

[191] Ueda H Shibahara N Takagi S Inoue T Katsuoka Y AST-120 treatment in pre-dialysis period affects the prognosis in patients on hemodialysis. Ren Fail (2008) 30:856–60. 10.1080/08860220802356531 18925523

[192] Noel S Martina-Lingua MN Bandapalle S Pluznick J Hamad ARA Peterson DA Intestinal microbiota-kidney cross talk in acute kidney injury and chronic kidney disease. Nephron Clin Pract (2014) 127:139–43. 10.1159/000363209 PMC4985236 25343838

[193] Rabb H Pluznick J Noel S The Microbiome and Acute Kidney Injury. Nephron (2018) 140:120–3. 10.1159/000490392 PMC6292672 29961049

[194] Lee T-H Park D Kim YJ Lee I Kim S Oh C-T Lactobacillus salivarius BP121 prevents cisplatininduced acute kidney injury by inhibition of uremic toxins such as indoxyl sulfate and pcresol sulfate via alleviating dysbiosis. Int J Mol Med (2020) 45:1130–40. 10.3892/ijmm.2020.4495 PMC7053870 32124946

[195] Andrade-Oliveira V Amano MT Correa-Costa M Castoldi A Felizardo RJF de Almeida DC Gut Bacteria Products Prevent AKI Induced by Ischemia-Reperfusion. J Am Soc Nephrol (2015) 26:1877–88. 10.1681/ASN.2014030288 PMC4520159 25589612

[196] Willing BP Russell SL Finlay BB Shifting the balance: antibiotic effects on host-microbiota mutualism. Nat Rev Microbiol (2011) 9:233–43. 10.1038/nrmicro2536 21358670

[197] Schulfer AF Battaglia T Alvarez Y Bijnens L Ruiz VE Ho M Intergenerational transfer of antibiotic-perturbed microbiota enhances colitis in susceptible mice. Nat Microbiol (2018) 3:234–42. 10.1038/s41564-017-0075-5 PMC5780248 29180726

[198] Jang HR Gandolfo MT Ko GJ Satpute S Racusen L Rabb H Early exposure to germs modifies kidney damage and inflammation after experimental ischemia-reperfusion injury. Am J Physiol Renal Physiol (2009) 297:F1457–65. 10.1152/ajprenal.90769.2008 PMC2781336 19675178

[199] Emal D Rampanelli E Stroo I Butter LM Teske GJ Claessen N Depletion of Gut Microbiota Protects against Renal Ischemia-Reperfusion Injury. J Am Soc Nephrol (2017) 28:1450–61. 10.1681/ASN.2016030255 PMC5407717 27927779

[200] McQuade JL Daniel CR Hess KR Mak C Wang DY Rai RR Association of body-mass index and outcomes in patients with metastatic melanoma treated with targeted therapy, immunotherapy, or chemotherapy: a retrospective, multicohort analysis. Lancet Oncol (2018) 19:310–22. 10.1016/S1470-2045(18)30078-0 PMC5840029 29449192

[201] Lennon H Sperrin M Badrick E Renehan AG The Obesity Paradox in Cancer: a Review. Curr Oncol Rep (2016) 18:56. 10.1007/s11912-016-0539-4 27475805 PMC4967417

[202] McQuade JL Daniel CR Helmink BA Wargo JA Modulating the microbiome to improve therapeutic response in cancer. Lancet Oncol (2019) 20:e77–91. 10.1016/S1470-2045(18)30952-5 PMC12908161 30712808

[203] Herbst RS Baas P Kim D-W Felip E Perez-Gracia JL Han J-Y Pembrolizumab versus docetaxel for previously treated, PD-L1-positive, advanced non-small-cell lung cancer (KEYNOTE-010): a randomised controlled trial. Lancet (Lond Engl) (2016) 387:1540–50. 10.1016/S0140-6736(15)01281-7 26712084

[204] Reck M Rodriguez-Abreu D Robinson AG Hui R Csoszi T Fulop A Pembrolizumab versus Chemotherapy for PD-L1-Positive Non-Small-Cell Lung Cancer. N Engl J Med (2016) 375:1823–33. 10.1056/NEJMoa1606774 27718847

[205] Wolchok JD Chiarion-Sileni V Gonzalez R Rutkowski P Grob J-J Cowey CL Overall Survival with Combined Nivolumab and Ipilimumab in Advanced Melanoma. N Engl J Med (2017) 377:1345–56. 10.1056/NEJMoa1709684 PMC5706778 28889792

[206] Lin S-Y Yang C-Y Liao B-C Ho C-C Liao W-Y Chen K-Y Tumor PD-L1 Expression and Clinical Outcomes in Advanced-stage Non-Small Cell Lung Cancer Patients Treated with Nivolumab or Pembrolizumab: Real-World Data in Taiwan. J Cancer (2018) 9:1813–20. 10.7150/jca.24985 PMC5968770 29805708

[207] Yan X Zhang S Deng Y Wang P Hou Q Xu H Prognostic Factors for Checkpoint Inhibitor Based Immunotherapy: An Update With New Evidences. Front Pharmacol (2018) 9:1050. 10.3389/fphar.2018.01050 30294272 PMC6159743

[208] Hugo W Zaretsky JM Sun L Song C Moreno BH Hu-Lieskovan S Genomic and Transcriptomic Features of Response to Anti-PD-1 Therapy in Metastatic Melanoma. Cell (2016) 165:35–44. 10.1016/j.cell.2016.02.065 26997480 PMC4808437

[209] Xu J Ma X Yu K Wang R Wang S Liu R Lactate up-regulates the expression of PD-L1 in kidney and causes immunosuppression in septic Acute Renal Injury. J Microbiol Immunol Infect (2019) S1684–1182(19):30168–9. 10.1016/j.jmii.2019.10.006 31727535

